# Fe^3+^-binding transferrin nanovesicles encapsulating sorafenib induce ferroptosis in hepatocellular carcinoma

**DOI:** 10.1186/s40824-023-00401-x

**Published:** 2023-07-01

**Authors:** Youmei Xiao, Zhanxue Xu, Yuan Cheng, Rufan Huang, Yuan Xie, Hsiang-i Tsai, Hualian Zha, Lifang Xi, Kai Wang, Xiaoli Cheng, Yanfeng Gao, Changhua Zhang, Fang Cheng, Hongbo Chen

**Affiliations:** 1 School of Pharmaceutical Sciences (Shenzhen), Shenzhen Campus of Sun Yat-Sen University, Shenzhen, 518107 People’s Republic of China; 2 Department of Pharmacy, The Seventh Affiliated Hospital of Sun Yat-Sen University, Shenzhen, China; 3 Department of Hepatobiliary Surgery II, ZhuJiang Hospital, Southern Medical University, Guangzhou, 510280 Guangdong Province China; 4 Department of Medical Imaging, The Affiliated Hospital of Jiangsu University, Zhenjiang, 212001 Jiangsu Province China; 5 Department of Pharmacy, Shenzhen Bao’an Maternal and Child Health Hospital, Shenzhen, 518133 Guangdong Province China; 6 Center for Digestive Disease, The Seventh Affiliated Hospital, Sun Yat-Sen University, Shenzhen, 518107 Guangdong Province China

**Keywords:** Transferrin, Biomembrane-based nanovesicles, Sorafenib, Ferroptosis, Hepatocellular carcinoma, Combination therapy

## Abstract

**Background:**

Ferroptosis, iron-dependent cell death, is an established mechanism for cancer suppression, particularly in hepatocellular carcinoma (HCC). Sorafenib (SOR), a frontline drug for the treatment of HCC, induces ferroptosis by inhibiting the Solute Carrier family 7 member 11 (SLC7A11), with inadequate ferroptosis notably contributing to SOR resistance in tumor cells.

**Methods:**

To further verify the biological targets associated with ferroptosis in HCC, an analysis of the Cancer Genome Atlas (TCGA) database was performed to find a significant co-upregulation of SLC7A11 and transferrin receptor (TFRC), Herein, cell membrane-derived transferrin nanovesicles (TF NVs) coupled with Fe^3+^ and encapsulated SOR (SOR@TF-Fe^3+^ NVs) were established to synergistically promote ferroptosis, which promoted the iron transport metabolism by TFRC/TF-Fe^3+^ and enhanced SOR efficacy by inhibiting the SLC7A11.

**Results:**

In vivo and in vitro experiments revealed that SOR@TF-Fe^3+^ NVs predominantly accumulate in the liver, and specifically targeted HCC cells overexpressing TFRC. Various tests demonstrated SOR@TF-Fe^3+^ NVs accelerated Fe^3+^ absorption and transformation in HCC cells. Importantly, SOR@TF-Fe^3+^ NVs were more effective in promoting the accumulation of lipid peroxides (LPO), inhibiting tumor proliferation, and prolonging survival rates in HCC mouse model than SOR and TF- Fe^3+^ NVs alone.

**Conclusions:**

The present work provides a promising therapeutic strategy for the targeted treatment of HCC.

**Graphical abstract:**

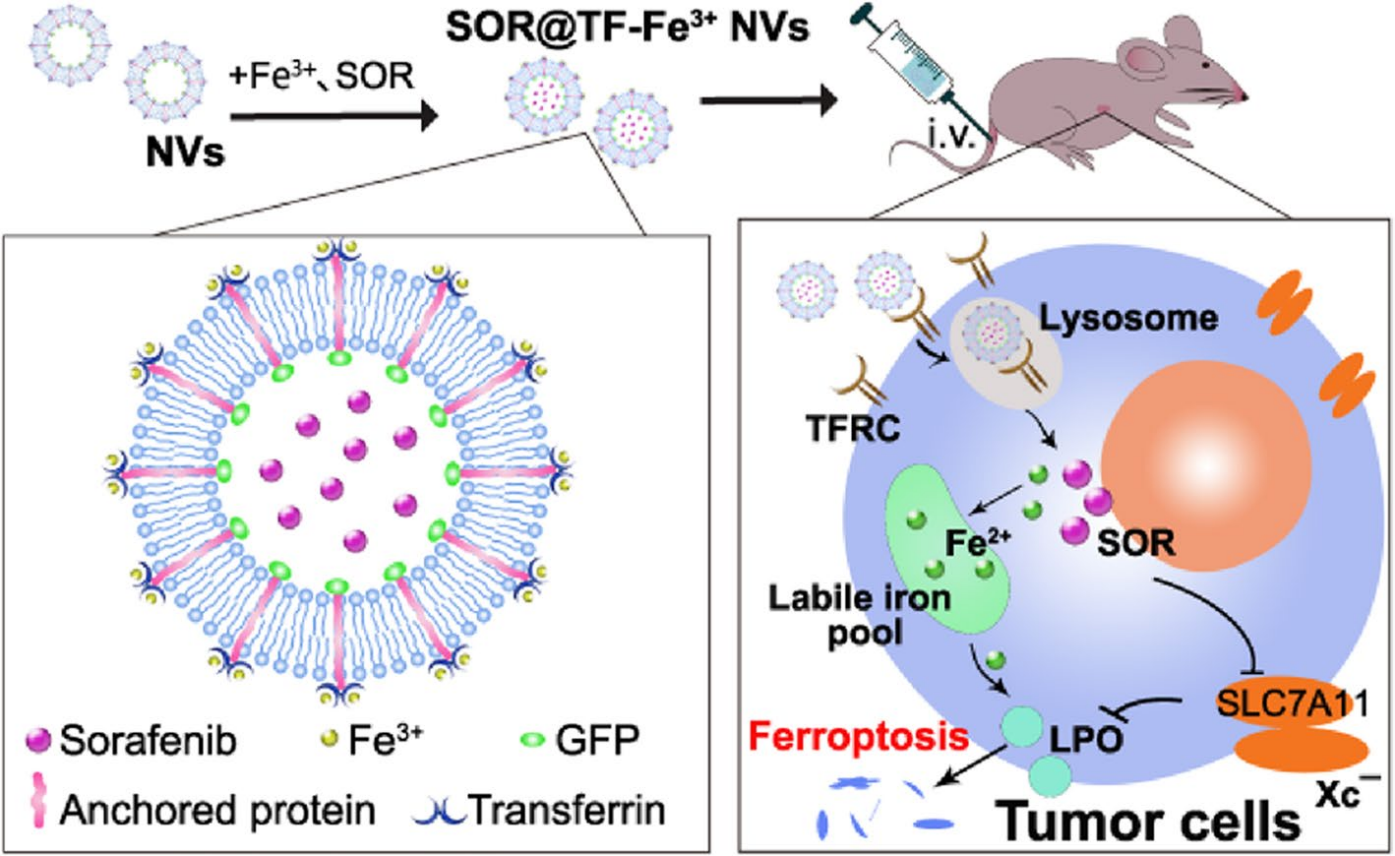

**Supplementary Information:**

The online version contains supplementary material available at 10.1186/s40824-023-00401-x.

## Introduction

As the most common form of liver cancer, Hepatocellular Carcinoma (HCC) is characterized by a high degree of malignancy and poor patient prognosis. Concerningly, the incidence of HCC is also increasing globally [1]. Typically, drugs for the treatment of HCC (*i.e.*, chemotherapeutics and other targeted therapies) are generally considered to induce cancer cell apoptosis for their anti-tumor response [2–4]. However, recent studies have shown that ferroptosis, a novel type of non-apoptotic cell death, plays a vital role in the occurrence and development suppression of HCC, and has the potential to serve as a novel target for HCC treatment [5–8].

Ferroptosis is believed to occur through the accumulation of membranous lipid peroxides, which are generated by reactive oxygen species (ROS), principally by a) the extrinsic or transporter-dependent pathway, e.g., decreased cysteine or glutamine uptake by SLC7A11 (Solute Carrier family 7 member 11) or SLC1A5 (Solute Carrier family 1 member 5) transporter, increased iron transport by TF (transferrin)—TFRC (transferrin receptor) pathway and decreased iron export by SLC40A1(Solute Carrier family 40 member 1) transporter, and b) the intracellular or enzyme-regulated pathway (e.g., the inhibition of glutathione peroxidase 4, GPX4). [9–11]. Among them, iron ion metabolism plays the most critical role. Normally, iron uptake is mediated by transferrin (TF, existing as the high affinity TF-Fe^3+^ complex in serum) which is recognized and absorbed into the cell by the TFRC. Bound Fe^3+^ is subsequently reduced to free Fe^2+^ though the STEAP3 metalloreductase in the endosome and released into the cytosol to constitute the labile iron pools (LIP) [12, 13]. In instances of high intracellular iron, however, over and above the capacity of the cell to quench ROS, irreversible lipid peroxidation can occur through classical Fenton pathways leading to general oxidative damage, and cell death [14–16]. This effect can be enhanced by the inhibition of the cystine/glutamate amino acid antiporter system X_C_¯, which leads to a decrease in the production of glutathione (GSH) – a key maintenance factor for the antioxidant activity of GPX4 [17, 18]. Notably, the FDA-approved drug sorafenib, a frontline treatment for HCC, has been shown to induce ferroptosis and suppress cancer cell growth though the inhibition of X_C_¯ [5, 6, 19].

Recently, evidence has emerged which suggests a link between SOR resistance in HCC and inadequate ferroptotic activity. For example, enhanced expression of the cysteine-rich metallothionein (MT)-1G protein, regulated by Nrf2 (a mediator of GPX4-related antioxidant process), facilitates SOR resistance by limiting lipid peroxidation [20]. In addition, a reduction in cytoplasmic Fe^2+^ content in tumor cells also limits the production of lipid ROS, thereby inhibiting ferroptosis and imparting nominal SOR resistance [21–23]. A deeper understanding of the molecular mechanism of ferroptosis-related SOR resistance would be incredibly valuable, and likely provide the foundation for effective HCC therapeutics. For that reason, we performed a search of ferroptosis-related factors in the TCGA-LIHC project from the Cancer Genome Atlas (TCGA) database and found that TFRC, a ubiquitously expressed membrane protein, and SLC7A11 (subunit of antiporter system X_C_¯) have significant upregulation (above other ferroptotic targets) in HCC cells when compared to normal tissue. Further analysis of our in-house clinical samples also confirmed this tendency.

**Scheme 1:**
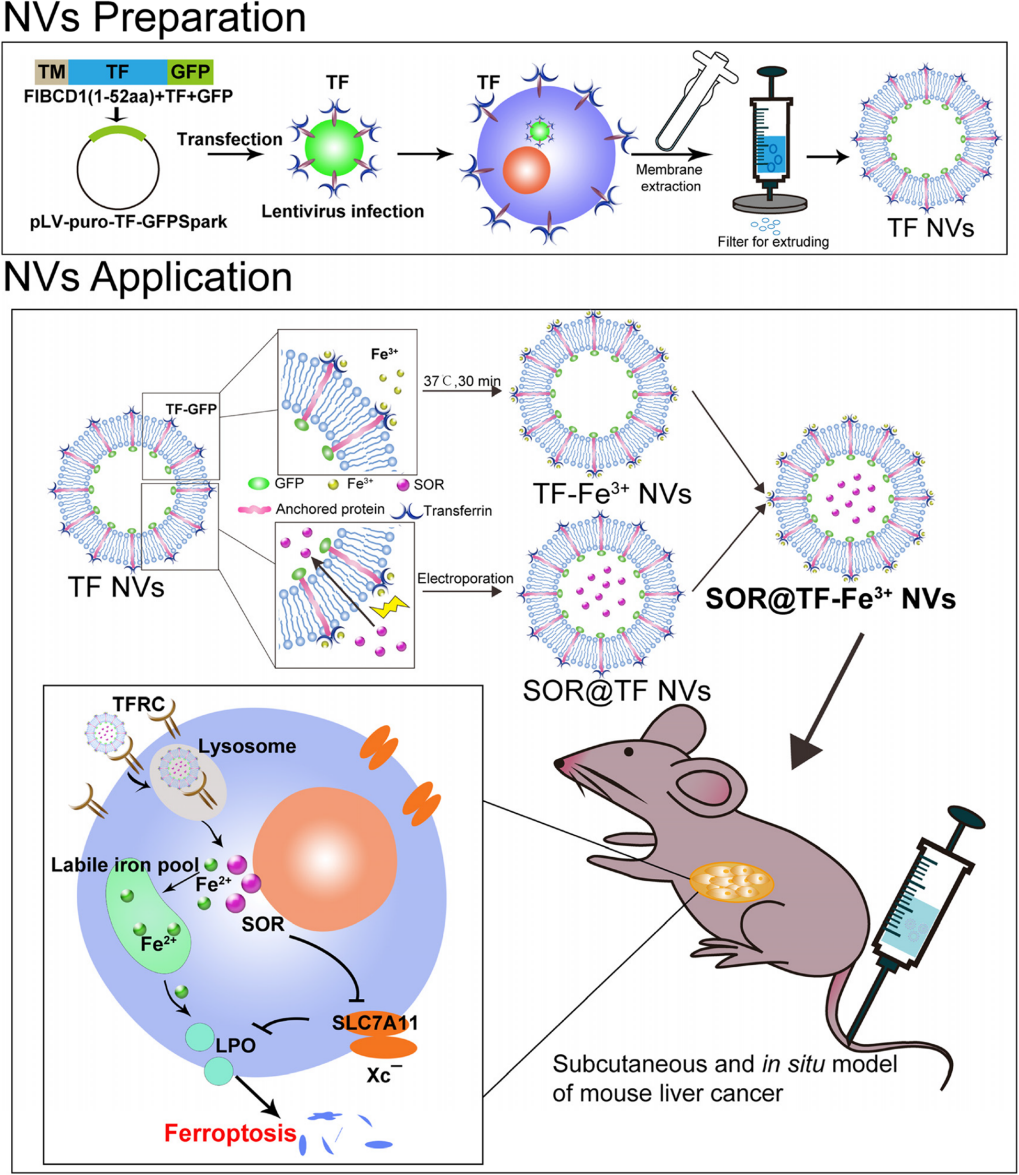
Graphical representation of SOR@TF-Fe^3+^NVs preparation and induced ferroptosis in HCC

In this study, we constructed cell membrane-derived nanovesicles bearing transferrin-Fe^3+^ (TF NVs) and encapsulated SOR (denoted as SOR@TF-Fe^3+^ NVs), which are expected to promote ferroptosis and overcome SOR resistance. We propose this is achieved by a dual mechanism of a) enhanced LIP accumulation by TFRC/TF-Fe^3+^, and b) inhibition of X_C_¯(SLC7A11) with concomitant reduction of antioxidant defense by SOR (Scheme 1). There are numerous advantages to the SOR@TF-Fe^3+^ NVs system, including straightforward preparation, and excellent biocompatibility [24, 25]. Furthermore, nano-sized TF NVs are expected to passively accumulate in liver tissue, and actively target TFRC-overexpressing HCC cells, thereby decreasing the risk of systemic Fe^3+^ and SOR delivery. Gratifyingly, in vivo and in vitro experiments demonstrated improved efficacy of SOR@TF-Fe^3+^ NVs in promoting ferroptosis and inhibiting HCC growth compared to SOR and TF-Fe^3+^ NVs alone. Therefore, SOR@TF-Fe^3+^ NVs may provide a promising therapeutic strategy for HCC treatment.

## Materials and methods

### Clinical samples

Clinical samples, including eight live cancer patients tissue samples, fifteen blood samples from normal volunteer, and fifteen blood samples from liver cancer patients respectively, were obtained from the Department of Hepatobiliary Surgery II in ZhuJiang Hospital of Southern Medical University. All patients gave informed consent for this study and all experiments were given by the Medical Ethics Committee of ZhuJiang Hospital of Southern Medical University, China (Approval number: 2022-KY-024–01).

### Chemicals and reagents

Sorafenib, Calcein-AM and TMRE were purchased from Med Chem Express (MCE). DCFH-DA (2'-7'-dichlorodihydrofluorescein diacetate) was purchased from Sigma. FeCl_3_**·**6H_2_O and NH_4_Cl were purchased from MACKLIN. Sulphosalicylic acid was purchased from Aladdin. Wheat germ agglutinin (WGA) Alexa Fluor 488 and 350 dyes were purchased from Thermo Scientific. GFP and OFP fluorescin antibodies for Western blotting were purchased from Transgen. COX2 antibodies for Western blotting and TFRC, Ki67 antibodies for IHC were purchased from Cell Signaling Technology.

### Plasmids and stable cell lines

Plasmids of PLV-puro-TF-GFPSpark and pLV-puro-TFRC-OFPSpark were synthesized by Sino Biological Inc. pLKO.1-U6-shTFRC-mcherry-puro was purchased from Kidan Biosciences. For stable cell lines, HEK 293T and HepG2 cells were infected with lentivirus, which were derived from target plasmids-transfected HEK-293T cells, and selected with puromycin (2 μg/mL).

### Cell line and cell culture

LO2 (human normal hepatocyte line) and Hepatoma cell lines, including HepG2, SMMC7721, Huh7, 7701, 7703 and 8103, were purchased from the American Type Culture Collection (ATCC). All cells were maintained in high glucose DMEM (Thermo Scientific, USA) with 100 U/mL penicillin, 0.1 mg/mL streptomycin (Genstar, China) and 10% (v/v) FBS, and cultured at 37 °C in a 5% CO_2_ incubator.

### Cell membrane nanovesicle preparation [26, 27]

HEK-293T cells stably expressing TF were cultured in 15 cm dishes with 10% DMEM, and allowed to proliferate to 80% confluence. After washing with cold PBS buffer (2 times), cells were collected with homogenization medium buffer (containing 0.25 M sucrose, 1 mM EDTA, 20 mM Hepes–NaOH, protease inhibitor cocktail, and adjusted to pH 7.4) and lysed overnight at 4 °C. Cell lysis suspensions were then milled with a serial extrusion tissue grinder on ice (200 times/mL). Following grinding, the entire solution was centrifuged at 5000 rpm for 10 min, and the collected supernatant centrifuged again at 12,000 rpm for 10 min. The precipitate was resuspended in PBS buffer, and filtered sequentially through 0.45 μm and 0.22 μm polycarbonate membrane filters to yield the final nanovesicle solutions. The concentration of NVs was quantified by bicinchoninic acid (BCA) kit.

### Size distribution and zeta-potential analysis

The size distributions and zeta potentials of NVs in PBS buffer were evaluated using a NanoBrook 90Plus PALS (Brookhaven instruments). Size distribution was quantified from 0 to 5000 nm. The average of three measurements obtained for data analysis.

### Nanovesicle morphology analysis

Drops of the appropriate solution of nanovesicles were pipetted directly onto a supporting film and incubated for 5 min. Uranium acetate solution (2%, 10 μL) was added to each droplet and incubated for a further 7 min in the dark. Following this, the supporting films were washed with ddH_2_O (2 times) and dried in air. Images were obtained from a transmission electron microscopy (TEM, hc-1, Hitachi) at 80 kV and the morphology of the NVs observed.

### Western blotting

Cells were initially lysed with Radio-immunoprecipitation assay (RIPA) lysis buffer (Thermo Scientific). The cell lysates and purified membrane vesicles were then purified through sequential separation on 10% SDS-PAGE and polyvinylidene fluoride membranes (Millipore, Darmstadt, Germany). After sealing with 5% non-fat milk (> 1 h at RT), primary antibodies for GFP, OFP, COX2, GPX4, β-actin, and Na^+^/K^+^-ATPase were incubated with the membranes overnight at 4 °C. Subsequently, horseradish peroxidase (HRP)-conjugated anti-rabbit or anti-mouse secondary antibodies were incubated with the membranes (1 h at RT). Finally, Enhanced Chemiluminescence Reagent (ECL, Protein Tech, China) was added and the plate imaged.

### SOR loading

2 mg (protein weight) TF NVs were gently mixed with 200 μg SOR in 1 mL cold PBS. The mixtures were added to 0.4 cm electroporation cuvettes and were subjected to electroporation at 300 V and 150 μF using a Bio-Rad Gene Pulser Xcell Electroporation System. Then, samples, with electroporation cuvettes, were incubated on ice for 30 min for the membrane recovery. Following centrifugation at 12 000 g for 10 min, the precipitated NVs were washed with cold PBS for 3 times and resuspended in cold PBS for further application.

### Ferric binding

100 μg ferric solution was mixed with 1 mg TF NVs (protein weight), incubated for 2 h at 37 °C, and then centrifugating at 12,000 rpm for 10 min. The supernatant was then mixed with 20% sulfosalicylic acid solution and NH_4_Cl-NH_3_ buffer solution successively, and the absorbance was detected by UV–Vis spectrophotometer at 425 nm for Ferric Binding Capacity test.

### Nanovesicle cell binding assay

293T-TFRC-OFP cells were seeded in confocal dishes and cultured at 37 °C in a 5% CO_2_ incubator. The next day, the membranes of cells were stained with WGA-ALEXA-350 for 10 min at 37 °C, washed with PBS buffer three times, and co-incubated with TF-GFP NVs (50 μg/mL, protein weight) for 30 min. Images were then acquired by the confocal microscopy (Zeiss, LSM880) in a 63 × objective.

### Establishment of SOR-resistant HCC cells

The HepG2 cells were grown in 6-well plates at 1 × 10^4^ cells/well and incubated with SOR at 2 μg/mL. Media changed every 3 days and a subculture performed when cells confluence reached 90%. The concentration of SOR was slowly increased by 1.5 μg/mL every two weeks. After 6 months, SOR-resistant HCC cell lines were obtained, named HepG2-SR.

### CCK-8 cell proliferation and cytotoxicity assay

5 × 10^3^ cells per well (HepG2, SMMC7721, HepG2-SR) were grown in 96-well plates with three wells used for each assayed group for the next day. After treatment for 24 h, cell numbers were evaluated through a cell counting kit-8 (CCK-8) with 10 μL/well. After the plate was incubated at 37 °C for 1 h, the absorbance (OD) value of each well was measured at 450 nm using a microplate analyzer (Victor Nivo Multimode Plate Reader).

### Colony-forming unit assays

The HCC cells (HepG2, SMMC7721, HepG2-SR) were cultivated in 6-well plates with 1000 cells per well for each experimental group. Cells were incubated for 10–14 d at 37 °C in 5% humidified CO_2_, until obvious cell clusters were observable. The clusters were then washed with 37 °C PBS buffer before being fixed with 4% paraformaldehyde, dyed with 0.5% crystal violet dye for 10 min, gently washed with distilled water, and dried. The number of colonies was then counted manually.

### Analysis of ferrous ion in cell

The HepG2 cells were collected and mixed with 4–10 times volume of Iron Assay Buffer (ab83366; Abcam), lysed by ultrasonication, and centrifuged at 12,000 rpm for 10 min, with the supernatant being collected. Supernatant samples were then incubated with an equal volume of Iron Reducer Buffer and Iron Probe Buffer at 37 °C for 1 h, and immediately detected by All-band microplate reader (Epoch2, Biotek) at 593 nm.

### Intracellular ROS analysis

Cells (HepG2, SMMC7721, HepG2-SR) in each experimental group were treated with 10 μmol/mL DCFH-DA (HY-D0940; MedChemExpress) for 20 min at 37 °C (shielded from light) and then washed with PBS buffer three times. The cells in confocal dishes were then viewed with confocal microscopy to assess the localization of DCFH-DA, and the relative fluorescent intensity of cells in 6-well plates were measured by flow cytometry.

### Mitochondrial membrane potential detection

Cells (HepG2, SMMC7721, HepG2-SR) were cultivated in 6-well plates, with 1 × 10^5^ cells per well for each experimental group. These were then collected by trypsinization, washed with PBS buffer by ultracentrifugation (1000 rpm, 5 min), and incubated with 150 nM TMRE (HY-D0985A; MedChemExpress) for 5 min in dark. Immediately following this, the mitochondrial membrane potential was measured by flow cytometry with no less than 10,000 cells per sample.

### Lipid peroxidation (MDA) assay

Cells (HepG2, SMMC7721, HepG2-SR) were cultivated in 10 cm dishes and allowed to proliferate to 80% confluence for each experimental group for 24 h treatment, which were then collected by trypsinization, washed with PBS by ultracentrifugation (1000 rpm, 5 min), and resuspended in PBS on ice (~ 4 °C). Following ultrasonication and centrifugation at 12,000 rpm for 10 min, cell-lysed supernatant was mixed with the appropriate amount of thiobarbituric acid solution as per the Lipid Peroxidation (MDA) Assay Kit (ab118970; Abcam) and incubated for 60 min at 95 °C in metal heater. After that, samples were cooled on ice for 10 min, and then were transferred to wells of microplate to analyze with microplate reader.

### Animal models

Approval for the use of laboratory animals and all animal experiments was given by the Animal Ethics Committee of Sun Yat-sen University, China (Approval number: SYSU-IACUC-2020–000375). Six- to eight-week-old specific pathogen-free female BALB/c nude mice were provided by SYSU Laboratory Animal Center, and maintained in an isolated SPF room held at a regulated temperature (25 ± 2 °C) and humidity (approximately 40–50%). The mice were housed under a 12 h/12 h light/dark cycle and fed with a clean diet and water.

#### Model of subcutaneously tumors

All protocols were approved and performed according to the guidelines of the Ethics Committee of SYSU. HepG2 cells were collected in the exponential phase and digested to form single-cell suspensions, with the concentration subsequently adjusted to 5 × 10^7^ cells/mL. Nude mice (35 mice), equally divided into 7 sub-groups, were disinfected with 75% alcohol and inoculated with 200 μL of HepG2 cell suspension by subcutaneous injection at the back of the neck (defined as day 0). Tumors were observed to reach a size of 60–70 mm^3^ after one week. On day 7, the appropriate treatment [*i.e.*, free NVs (293T NVs, 25 mg/kg), Fe^3+^ solution (3 mg/kg), TF NVs (25 mg/kg), TF-Fe^3+^ NVs (25 mg/kg), SOR (5 mg/kg), SOR@TF NVs (25 mg/kg), or SOR@TF-Fe^3+^ NVs (25 mg/kg)] were administered into mice by tail-vein injection every other day (*i.e.*, day 7, day 9, etc.). All mice were monitored and tumor size was calculated every day, with tumor volume calculated as V = d^2^ × D/2 (with D and d as the maximum and minimum diameter of tumor respectively) [26]. Meanwhile, mice were monitored for weight loss to assess potential toxicities. Euthanasia measures were applied when animals exhibited signs of impaired health, or tumor volume that exceeded 2 cm^3^.

#### In situ model of mouse liver cancer

HepG2 cells were digested by trypsin and adjusted to 5 × 10^7^ cells per 50 μL. Briefly, single-cell suspensions and PBS (50 μL, respectively) were injected into the left liver of nude mice (5 weeks old, weighing 18-22 g) after anesthetizing. Five days after the in situ HCC models were established, mice were treated with free NVs (25 mg/kg), SOR (5 mg/kg), SOR@TF-Fe^3+^ NVs (25 mg/kg) by tail-vein injection every other day. All mice were monitored for weight every other day. Euthanasia measures were applied when animals exhibited signs of impaired health.

### Hematoxylin–eosin staining

Tumor and other tissue (liver, spleen, kidney, heart and lung), collected from different treatments, were fixed in 4% paraformaldehyde for 24 h and paraffin embedded. Sections (4 μm thickness) were stained with H&E using the standard procedure. The H&E staining images were observed by microscopy with 10 × magnification.

### Immunohistochemistry (IHC)

Immunohistochemistry was performed to confirm the expression of TFRC or Ki67 in liver patients and tumor models. All tissue samples were subjected to deparaffinization, rehydration, and heat-induced epitope retrieval and were subsequently incubated with TFRC or Ki67 primary antibodies overnight. Following overnight incubation, secondary antibodies were dropped to tissue sections and again incubated at room temperature in the dark for 1 h. Subsequently, sections were washed with PBS and observed by microscope with 10 × magnification.

### Prussian blue staining

Dewaxed tissue samples were co-incubated with Perls’ stain solution at room temperature for 30 min in the dark, and then washing with ddH_2_O (3 min). After staining with the redyeing solution of nuclear solid red (10 min), and washing again (5 min), the tissue samples were observed under a microscope.

### Quantitative real-time PCR

The total RNA was collected from cells or tumor tissue using TRIZOL reagent (TaKaRa, Tokyo, Japan), then cDNA (complementary DNA) was synthesized using HiScript III RT SuperMix for qPCR (+ gDNA wiper) (TransGen Biotech, China) by PCR Instrument (BIO-RAD). Subsequently, 2 × SYBR Green qPCR Mix (TransGen Biotech, China) was used to quantify the relative gene expression, which was performed by LightCycler 96. The folding changes of mRNA were identified by 2^–ΔΔCt^ method. The qPCR primers for all experiments are listed in Supplementary Table 1.

### TCGA database analysis

The FPKM-normalized RNA-seq (RNA-sequencing) data and corresponding clinical data of patients in the TCGA-LIHC project from the TCGA database were downloaded from TCGA official website (https://tcga-data.nci.nih.gov/tcga/). The data of 50 normal samples and 374 cancer patients were obtained for relative gene expression analysis, especially regarding GPX4 (Ensembl_ID: ENSG00000167468.15), SLC1A5 (Ensembl_ID: ENSG00000105281.11), SLC40A1 (Ensembl_ID: ENSG00000138449.9), SLC7A11 (Ensembl_ID: ENSG00000151012.12), TF (Ensembl_ID: ENSG00000091513.13), TFRC (Ensembl_ID: ENSG00000072274.11) were analyzed. Two-tailed paired Student’s t-test was used for statistical analysis.

### Statistical analysis

All experiments were carried out independently at least three times unless stated otherwise. Data analysis was performed using GraphPad Prism (Version 7.0, GraphPad Software Inc., USA). One-way analysis of variance (ANOVA) and Tukey’s test were conducted as noted in the text, with *P* < 0.05 being considered the threshold for statistically significance (*P ≤ 0.05; **P ≤ 0.01; ***P ≤ 0.001). Data were expressed as means ± SEM.

## Results

### The co-upregulation of SLC7A11 and transferrin receptor (TFRC) in HCC samples

In order to data-mine for ferroptosis-related molecular changes, 425 RNA sequences (including 374 HCC samples and 50 normal samples) were obtained from the Cancer Genome Atlas (TCGA), and the expression levels of several key ferroptosis markers were analyzed. As noted previously, the cyst(e)ine/GSH- GPX4 axis is one of the most important mechanisms for ferroptosis defense, and ferroptosis can be triggered by inhibition of the upstream regulator system X_C_¯ cystine-glutamate antiporter or inactivation of the downstream effector GPX4 of GSH [17, 18]. Thus, we first evaluated the expressions of GPX4, SLC7A11 (also known as xCT [28], a subunit of the glutamate/cystine antiporter X_C_¯ system) and SLC1A5 (a high-affinity glutamine transporter). As expected, all showed significantly enhanced expression, which suggested to us that blocking of the GSH-GPX4 axis in HCC may promote ferroptosis and suppress tumor growth. This naturally suggested the use of the multi-kinase inhibitor SOR (approved for the treatment of HCC) given reports of it targeting of X_C_¯(SLC7A11) to trigger ferroptosis [19, 29]. However, other studies have shown that sole inhibition of the X_C_¯(SLC7A11)-GPX4 pathway by SOR does not sufficiently induce ferroptosis in HCC, and can even engender drug resistance [30, 31]. To overcome this, we hypothesized that a combination therapy which engaged multiple molecular targets [not just X_C_¯(SLC7A11)] may be a more effective strategy for HCC treatment.

**Fig. 1:**
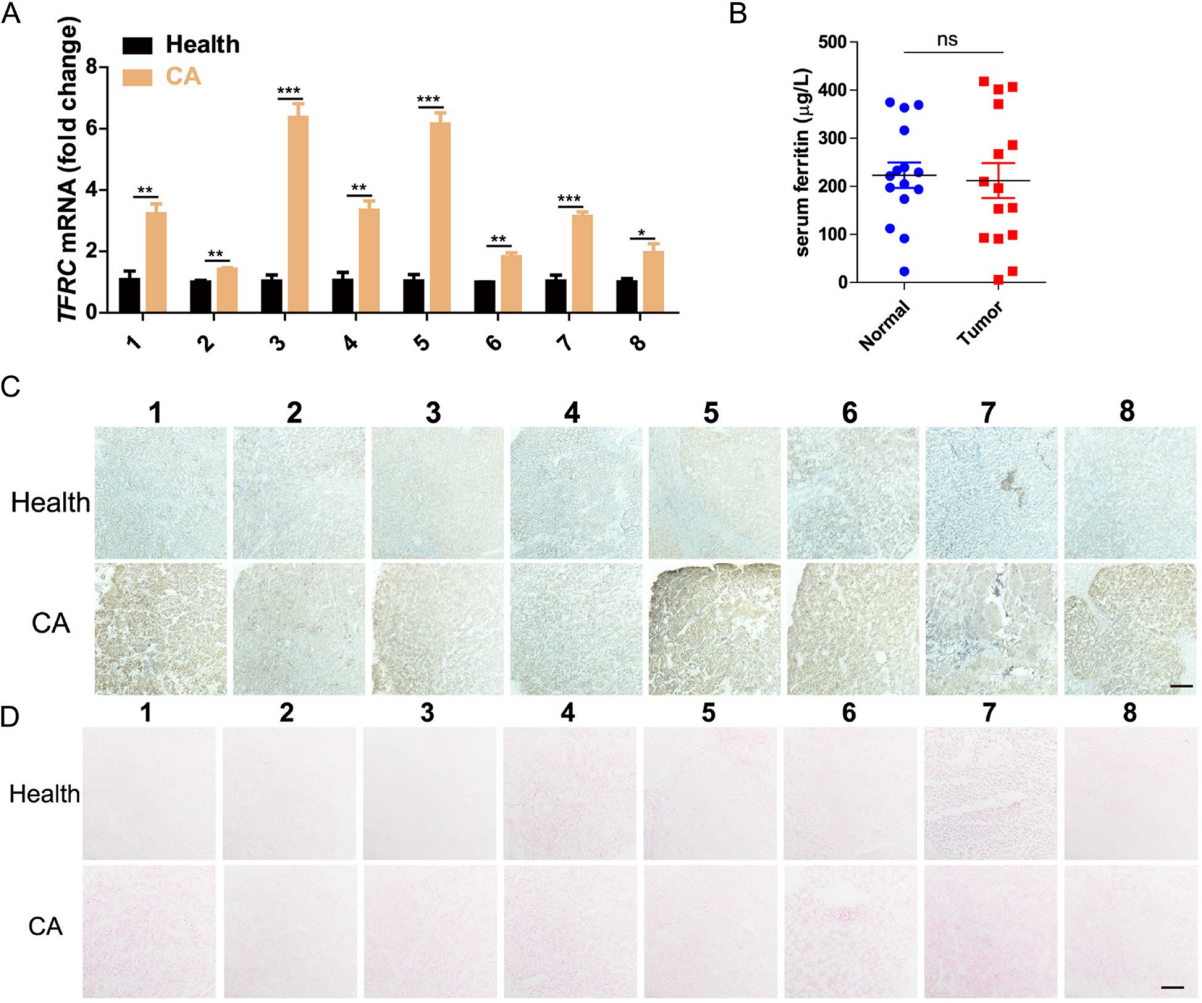
The expression level of TFRC and transferrin in clinical samples. **A** The expression level of TFRC in the cancer nest area (CA) and healthy liver tissue (Health) more than 1 cm away from the CA of 8 clinical liver cancer patients was detected by qPCR. Data are expressed as mean ± standard error (SEM), *n* = 3. *P ≤ 0.05, **P ≤ 0.01, ***P ≤ 0.001, one-way analysis of variance, ANOVA. **B** The serum transferrin level between non-tumor patients (15 cases) and tumor patients (15 cases) were detected. ns: no significant, one-way analysis of variance, ANOVA. **C**, **D** Representative images of TFRC immunohistochemistry and Prussian blue staining for clinical samples. Scale bar: 100 μm

Understandably, the process of ferroptosis is intimately linked to cellular iron metabolism, including uptake, conversion, utilization, and export. Notably, our genomic analysis determined that there was no significant difference in expression level of ferroportin [FPN, SLC40A1 (solute carrier family 40, member 1)], a transmembrane iron export protein, between HCC patients and normal group (Figure S1A, Supporting Information). In marked contrast, the TFRC in HCC patients was found to be significantly more abundant in both the TCGA samples (Figure S1A, Supporting Information) and our 8 clinical HCC cases (Fig. 1A, C and Figure S1B, Supporting Information). We therefore speculated that the high expression of iron-importing TFRC, but not iron-exporting ferroportin, could promote the accumulation of iron in HCC cells. However, Prussian blue staining showed no significant difference in iron accumulation between HCC liver tissues and healthy tissue (Fig. 1D), which indicated that simply increasing the expression of receptor TFRC in HCC cells could not effectively promote the accumulation of iron ions. This result was confirmed by further experiments demonstrating that there was no significant difference in iron accumulation between TFRC-knockdown, -overexpression and wild-type HCC transplanted tumors (Figure S2, Supporting Information). We further investigated the expression level and serum content of transferrin, the ligand of TFRC, in TCGA samples and our clinical samples, respectively. In contrast to TFRC, the expression level of transferrin decreased significantly in TCGA HCC patients (Figure S1A, Supporting Information). No significant difference in serum ferrintin was observed between HCC patients and healthy donors (Fig. 1C). Thus, the low-levels of transferrin expression and limited-ferrintin level may be an important reason for the undifferentiated accumulation of iron ions in TFRC high-expressed HCC and maintenance of iron homeostasis. However, it also suggested the tantalizing prospect that supplementation with exogenous transferrin-Fe^3+^ complex may promote the specific accumulation of iron ions in HCC. Such an analysis strongly suggested that a combined strategy of inhibiting the GSH- GPX4 axis coupled with substantial transferrin/iron dosing could provide an integrated strategy to promote the ferroptotic sensitivity in HCC cells.

### Establishment and characterization of SOR@TF-Fe^3+^ double-target NVs

**Fig. 2:**
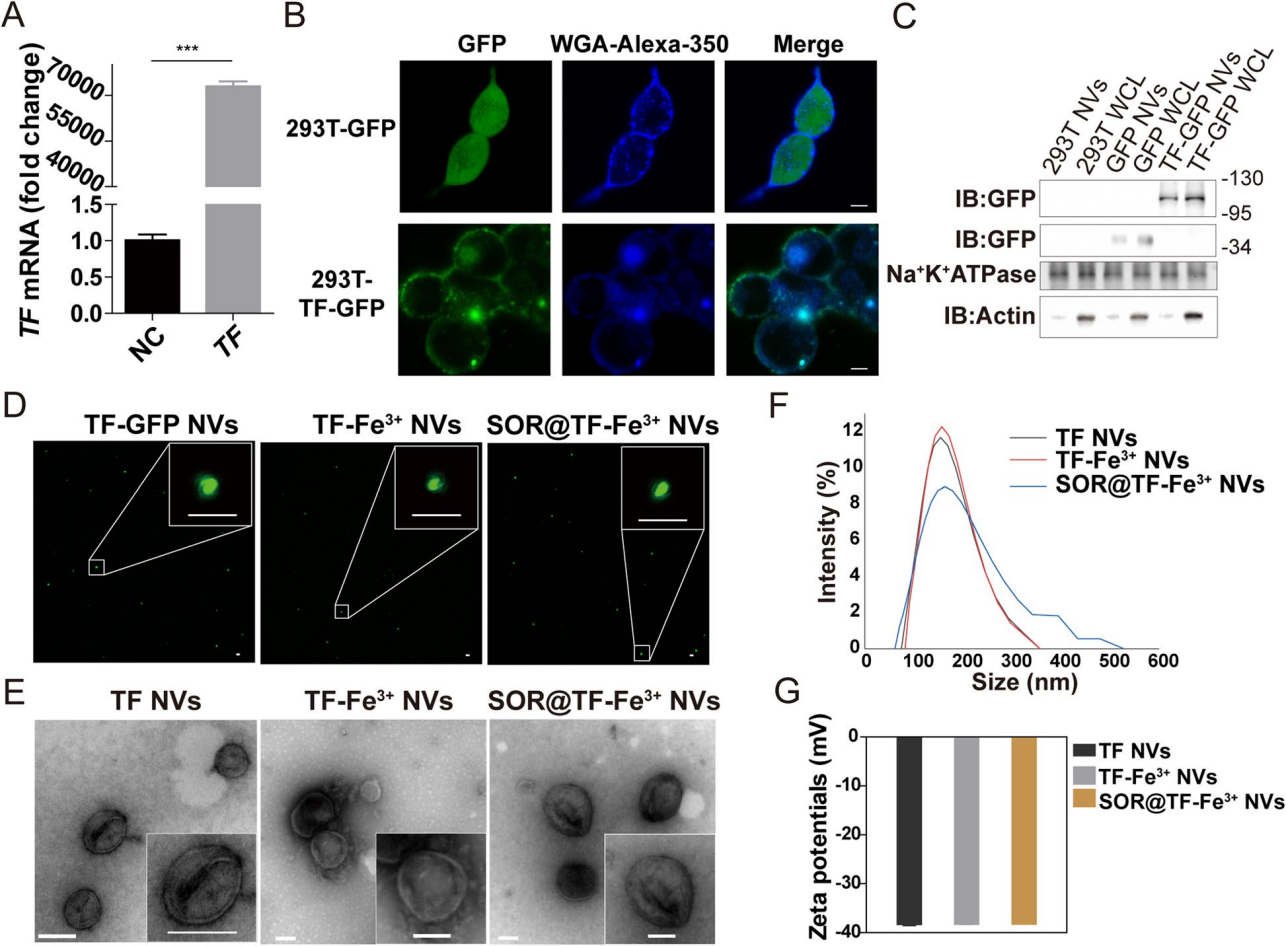
Construction and characterization of TF NVs, TF-Fe^3+^ NVs and SOR@TF-Fe^3+^ NVs. **A**, **B** qPCR results (*n* = 3, ***P ≤ 0.001, one-way analysis of variance, ANOVA.) and confocal images (Scale bar: 5 μm) showed the 293T-TF-GFP cells were constructed. **C** Western blot analysis of GFP protein label expression in 293T cells, 293T NVs, 293T-GFP cells, 293T-GFP NVs, 293T-TF-GFP cells, 293T-TF-GFP NVs protein lysate, WCL: whole cell lysate. **D** The green spots were observed under confocal. Scale bar: 2 μm. **E** The morphology of the nanovesicles was observed by transmission electron microscopy (TEM), Scale bar: 100 nm. **F** Dynamic light scattering (DLS) analysis of the size distribution of TF NVs, TF-Fe^3+^ NVs and SOR@TF-Fe^3+^ NVs. **G** Phase analysis scattering (PALS) was used to analyze the Zeta potential distribution of all type NVs, *n* = 3

Due to its excellent biocompatibility, protein display capacity and drug loading ability, cell membrane-based nanovesicles (NVs) have recently become a promising drug delivery system [24]. Here, to sensitize TFRC highly-expressed HCC cells to ferroptosis induction, a nanovesicle simultaneously displaying high levels of Fe^3+^-loaded transferrin and encapsulating SOR was prepared. Briefly, HEK293T cells were infected by lentivirus carrying a membrane-localized form of GFP-transferrin. qPCR (Fig. 2A) and confocal images (Fig. 2B) cooperatively confirmed the high expression and its cell membrane localization of exogenous GFP-transferrin. Next, cell membrane-based transferrin nanovesicles (TF NVs) derived from the HEK293T cells with stable TF overexpression were prepared and purified though differential centrifugation, and an abundant presentation of GFP-transferrin protein on the purified NVs compared to the whole cell lysate of HEK-293T-TF cells was corroborated by western blotting analysis (Fig. 2C). Subsequently, TF NVs were co-incubated with Fe^3+^ at 37 °C for 24 h to prepare TF-Fe^3+^ NVs [32], with a detected binding rate of 90% (Figure S3A, Supporting Information). Furthermore, synthesis of SOR@TF-Fe^3+^ NVs with a 35% encapsulation efficiency of SOR (Figure S3B, Supporting Information) was also achieved by the electro-transfection method [26]. We subsequently tested whether the loading of Fe^3+^ and the encapsulation of SOR would affect the characterization of TF NVs. Confocal images (Fig. 2D) and transmission electron microscopy (TEM, Fig. 2E) showed the similar round shapes, TF localization, bi-molecular membrane structure and size distribution regardless of whether they were bound with iron ions or encapsulated with SOR. Moreover, dynamic light scattering (DLS) and phase analysis light scattering (PALS) further verified similar size and stability of these NVs, which had an average diameter of 158 nm and average zeta potential of -38 mV (Fig. 2F-G). Overall, the above results confirmed that SOR@TF-Fe^3+^ NVs were successfully prepared from engineered HEK-293T-TF cells and suitable for subsequent use.

### SOR@TF-Fe^3+^ NVs have good liver distribution and targeting of HCC cells with high expression of TFRC receptor, and can specifically promote the accumulation of iron and SOR in HCC cells

**Fig. 3:**
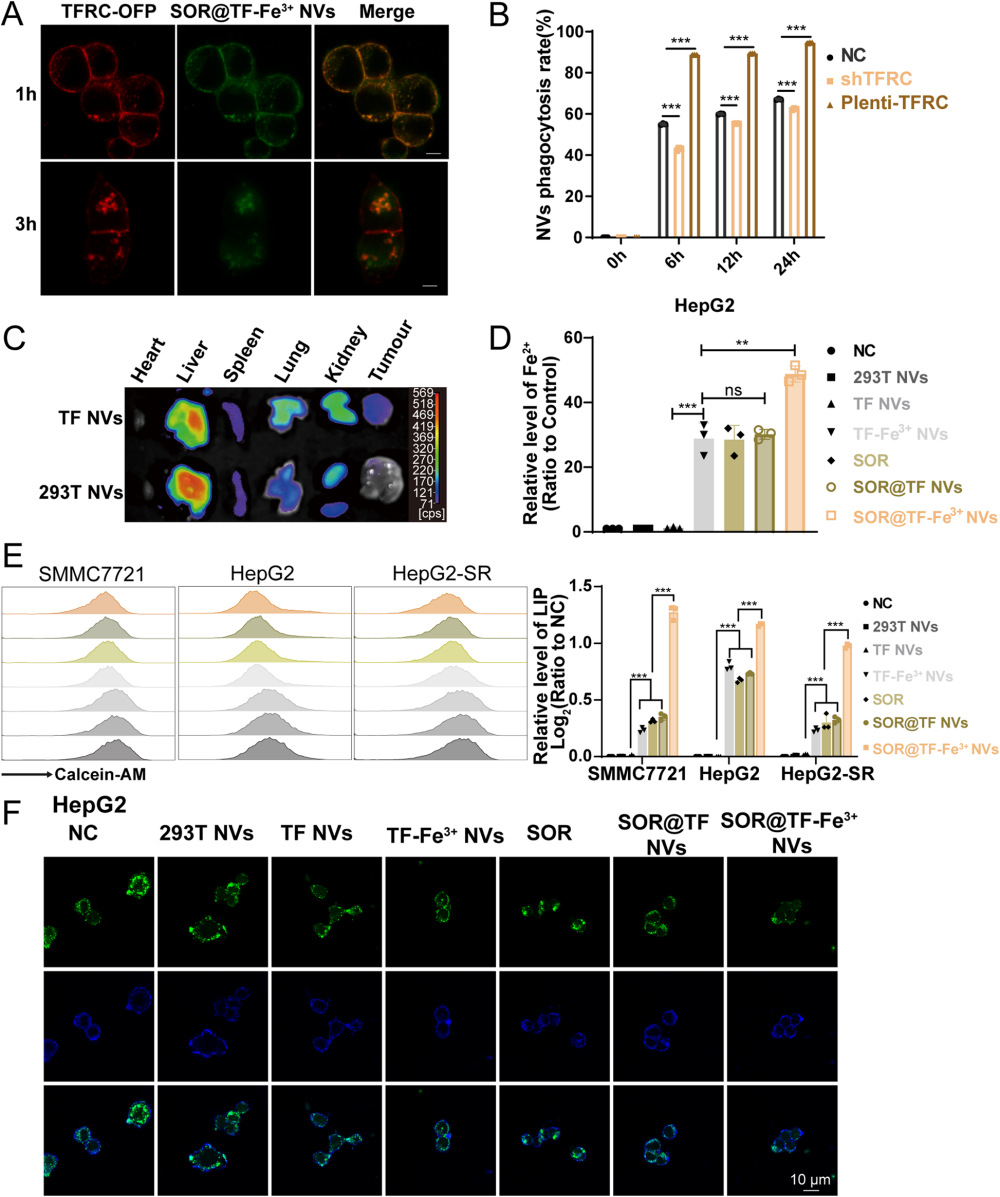
In vitro biological behavior and functions of SOR@TF-Fe^3+^ NVs. **A** SOR@TF-Fe^3+^ NVs were co-incubated with OFP-TFRC-expressing HEK-293T cells for 1 h or 3 h at 37 °C. The confocal image showed the co-localization of TF-engineered NVs labeled with green fluorescence and TFRC film expressing red fluorescence. Scale bar: 5 μm. **B** Flow cytometry was used to analyze the binding rates of TF-Fe^3+^ NVs (50 μg NVs/mL) between TFRC-knockdown, -overexpression and wild-type HepG2 cells at 0, 6, 12 and 24 h. **C** The distribution of TF NVs and 293 T NVs labeled by Cyanine 5.5 NHS ester in mice was detected by in vivo imager. **D** HepG2 cells in each experimental group were treated for 24 h, and the content of iron divalent ion in cells was detected by iron ion detection kit. **E**–**F** HCC cells were stained with Calcein-AM (final concentration:50 nΜ) and the cellular LIP level was analyzed by flow cytometry (**E**) and confocal images (**F**). Data are expressed as mean ± standard error (SEM), *n* = 3, ns: no significant, **P ≤ 0.01, ***P ≤ 0.001, one-way analysis of variance, ANOVA

Given that Fe^3+^-loaded transferrin targets TFRC for internalization and absorption of Fe^3+^ [15], we were eager to investigate whether SOR@TF-Fe^3+^ NVs were able to effectively bind TFRC and enter cells. Confocal microscopy (Fig. 3A) showed a distinct colocalization of TF-GFP and its receptor TFRC-OFP on the cell membranes as early as 1 h following co-incubation of SOR@TF-Fe^3+^ NVs with HEK293T cells stably expressing TFRC-OFP (detected by qPCR and Western blot in Figure S4, Supporting Information). What is more, with a corresponding extension of incubation time, the amount of endocytosis of TF-GFP and TFRC-OFP significantly increased in TFRC-OFP over-expressed HEK293T cells (Fig. 3A). To determine the effect of receptors on endocytosis, flow cytometry was used to investigate the endocytosis efficiency of wild-type, TFRC-knockdown and -overexpressed HepG2 cells on TF-Fe^3+^ NVs at 0, 6, 12, and 24 h after co-incubation. As shown in Fig. 3B, exogenous expression of TFRC significantly promoted the phagocytosis ability of HepG2 cells to TF-expressing vesicles, while TFRC knockdown markedly attenuated its phagocytosis efficiency, suggesting that the phagocytosis of HepG2 on TF-expressing vesicles is at least partially dependent on TF/TFRC interaction. This was further confirmed by in vivo bio-distribution studies of TF-expressed NVs and TF-free NVs administered by injection into mouse tail veins, with TF NVs showing stronger retention signals in TFRC-expressing tumors when compared to free NVs (Fig. 3C). In addition, it was observed that nano-sized NVs could passively accumulate in liver tissue, likely due to the enhanced permeability and retention (EPR) effect (Fig. 3C). These characteristics further underscore the potential of SOR@TF-Fe^3+^ NVs as a targeted therapy for liver cancer patients.

As noted previously, the cytoplasmic LIP is predominantly composed of free Fe^2+^ (formed from the reduction of Fe^3+^), and indeed this form serves as a putative biomarker of ferroptosis [16, 33]. We therefore measured the intracellular Fe^2+^ content in the different experimental groups through an iron assay kit. Consistent with expectation, HepG2 cells treated with TF-Fe^3+^ NVs had higher levels of Fe^2+^ accumulation than the free NVs or TF NVs, confirming that TFRC could mediate the targeted delivery of Fe^3+^-binding NVs to HCC cells (Fig. 3D). It is also important to note, however, that free SOR, as well as SOR-containing TF NVs (*i.e.*, not containing Fe^3+^) also increased the cellular level of Fe^2+^. We suspect that this may be caused by inhibition of ferritin heavy chain 1 (FTH1), given that SOR has previously been observed to promote intracellular Fe^2+^ release by this pathway [19, 21]. However, SOR@TF-Fe^3+^ NVs treatment resulted in the most significant Fe^2+^ accumulation, indicating the synergistic effect of TF-Fe^3+^ and SOR on LIP levels. To corroborate these results, a calcein-AM based assay (calcein-acetoxymethyl ester, a fluorophore with quenching positively correlated to iron concentration) was used to quantify the intracellular LIP [16]. Flow cytometry (Fig. 3E) and confocal microscopy (Fig. 3F) showed TF-Fe^3+^ NVs, SOR and SOR@TF NVs all significantly reduced intracellular calcein-AM fluorescence compared to 293T NVs and TF NVs treatments, with SOR@TF-Fe^3+^ NVs inducing the strongest reduction in fluorescence levels.

To further verify the correlation between SOR resistance in HCC and the accumulation of intracellular LIP, SOR-resistant HepG2 cells were obtained. After incubation with 10 μg/mL of SOR for 24 h, the cell activity of HepG2-SR were 80.65%, which were significantly higher than that of HepG2 cells (68.1%; Figure S5A, Supporting Information). More interestingly, the intracellular calcein-AM fluorescence of HepG2-SR cells, compared to HepG2 cells, were reduced by 70.6% and 16.1% respectively under the treating of TF-Fe^3+^ NVs and SOR@TF NVs (Fig. 3E).

In summary, it was confirmed that SOR@TF-Fe^3+^ NVs could specifically promote the accumulation of iron and SOR in TFRC high-expressed HCC cells.

### SOR@TF-Fe^3+^ NVs induced a significant ferroptosis-dependent growth inhibition of HCC cells in vitro

**Fig. 4:**
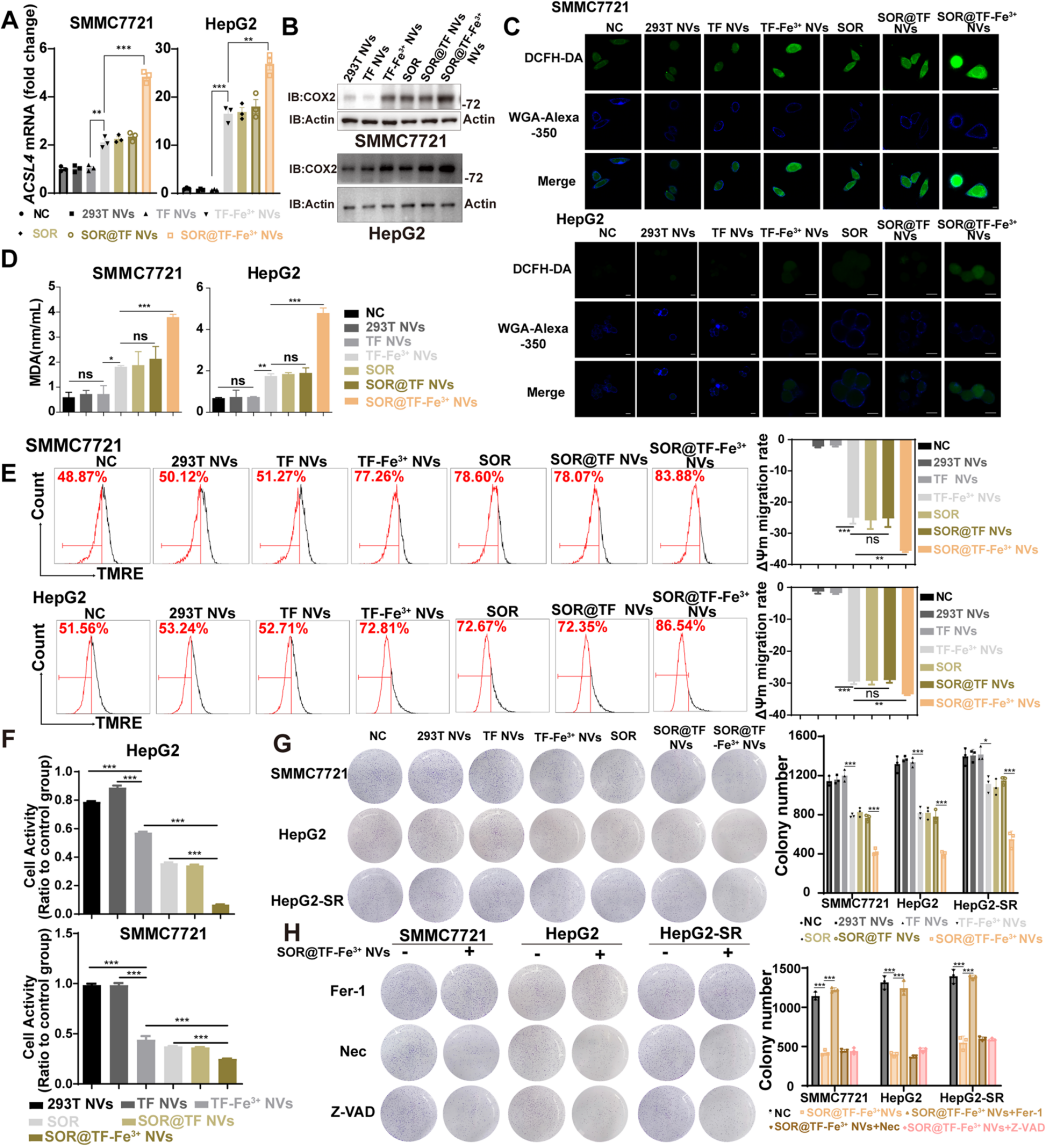
SOR@TF-Fe^3+^ NVs induced ferroptosis in HCC cells. **A** qPCR was used to analyze the expression of *ACSL4* mRNA. **B** Western blot analysis of COX2 protein expression. **C** The laser confocal image showed the ROS production after treatment for 24 h. Scale bar: 10 μm. **D** The content of MDA after treatment for 24 h was detected by the kit. **E** Flow cytometry analysis was used to test the change of ΔΨm. On the right is the histogram of curve migration quantitative analysis. **F** CCK-8 was used to detect the activity changes of HCC cells after 24 h in each treatment group. **G** The cell clone formation of SMMC7721, HepG2 and HepG2-SR cells in each experimental group was detected after 7 days of treatment, Dosage: SOR 1 μg/mL, nanovesicles 10 μg/mL (protein weight). **H** Cell growth was measured by clone formation assay when co-incubated with ferroptosis inhibitor Ferrostatin-1 (Fer-1, 2 μM), necrosis inhibitor Necrostatin 1 (Nec, 0.5 μM) and apoptosis inhibitor Z-VAD-FMK (Z-VAD, 2 μM). And the quantitative analysis of clone formation was shown on the right. Data are expressed as mean ± standard error (SEM), *n* = 3, ns: no significant, *P ≤ 0.05, **P ≤ 0.01, ***P ≤ 0.001, one-way analysis of variance, ANOVA

To investigate the effect of SOR@TF-Fe^3+^ NVs on ferroptosis, the expression levels of COX2 (Cyclooxygenase-2) and ACSL4 (Long-chain-fatty-acid-CoA ligase 4), two key enzymes in the lipid peroxide biosynthesis pathway which are accepted biomarkers of ferroptosis [34, 35], were detected by qPCR. The mRNA expression levels of ACSL4 and COX2 were significantly elevated in TF-Fe^3+^ NVs (with 2.3-fold change), SOR and SOR@TF NVs treated cells (SMMC7721, HepG2 and HepG2-SR) compared to 293T NVs and TF NVs groups, with the greatest increase observed in the SOR@TF-Fe^3+^ NVs-treated group (with 4.0-fold change, Fig. 4A, B and Figure S5B, Supporting Information). The most significant level of ROS accumulation was also identified in SOR@TF-Fe^3+^ NVs treated cells, as determined by DCFH-DA Staining (2,7-Dichlorodi -hydrofluorescein diacetate, Fig. 4C and Figure S6, Supporting Information) which detects malondialdehyde (MDA) generation (Fig. 4D and Figure S7A, Supporting Information), the principal product of polyunsaturated fatty acid peroxidation [31, 36, 37]. Excessive accumulation of ROS and MDA can cause cell damage, and so cytotoxicity was evaluated by measuring the mitochondrial membrane potential change (ΔΨm) using tetramethylrhodamine ethyl ester (TMRE). As expected, SOR@TF-Fe^3+^ NVs treated cells yielded the most significant dissipation of the ΔΨm (by 72% and 68% in SMMC7721 and HepG2 cells at 24 h, respectively) (Fig. 4E and Figure S7B, Supporting Information).

The growth and proliferation of SMMC7721 and HepG2 cells was further assessed by CCK-8 and colony formation assays. As shown in Fig. 4 F-G and Figure S7C, the treatments with TF-Fe^3+^ NVs, SOR and SOR@TF NVs significantly inhibited cell viability and colony formation, and SOR@TF-Fe^3+^ NVs showed the strongest inhibitory effect. In order to confirm that ferroptosis specifically was responsible for the observed TF-Fe^3+^ NVs-induced growth inhibition (and not general apoptotic or necrotic processes), HCC cells were co-treated with TF-Fe^3+^ NVs and the ferroptosis inhibitor ferrostatin 1, the necrosis inhibitor necrostatin 1 or the apoptosis inhibitor Z-VAD-FMK respectively, with colony formation observed by hematoxylin–eosin staining. Notably, only ferrostatin-1 was able to significantly ameliorate TF-Fe^3+^ NVs induced cell death (Fig. 4H), which further indicated that SOR@TF-Fe^3+^ NVs explicitly induce ferroptotic growth inhibition of HCC cells. Although inadequate ferroptosis occurred in SOR-resistant HepG2 cells compared to parental HepG2, SOR@TF-Fe^3+^ NVs showed the significant difference in promoting ferroptotic activity compared to control group.

Taken together, these results strongly support the notion that SOR@TF-Fe^3+^ NVs are targeted initiators of HCC cell ferroptosis, given the enhanced presence of validated biomarkers (COX2, ACSL4, as well as Fe^2+^), the marked accumulation of lipid peroxides ROS and MDA products, evidence of mitochondrial membrane damage, and indeed eventual cell death except in the presence of ferroptotic inhibitors (ferrostatin 1).

### SOR@TF-Fe^3+^ NVs significantly suppressed the growth of HCC xenograft tumors in vivo

**Fig. 5:**
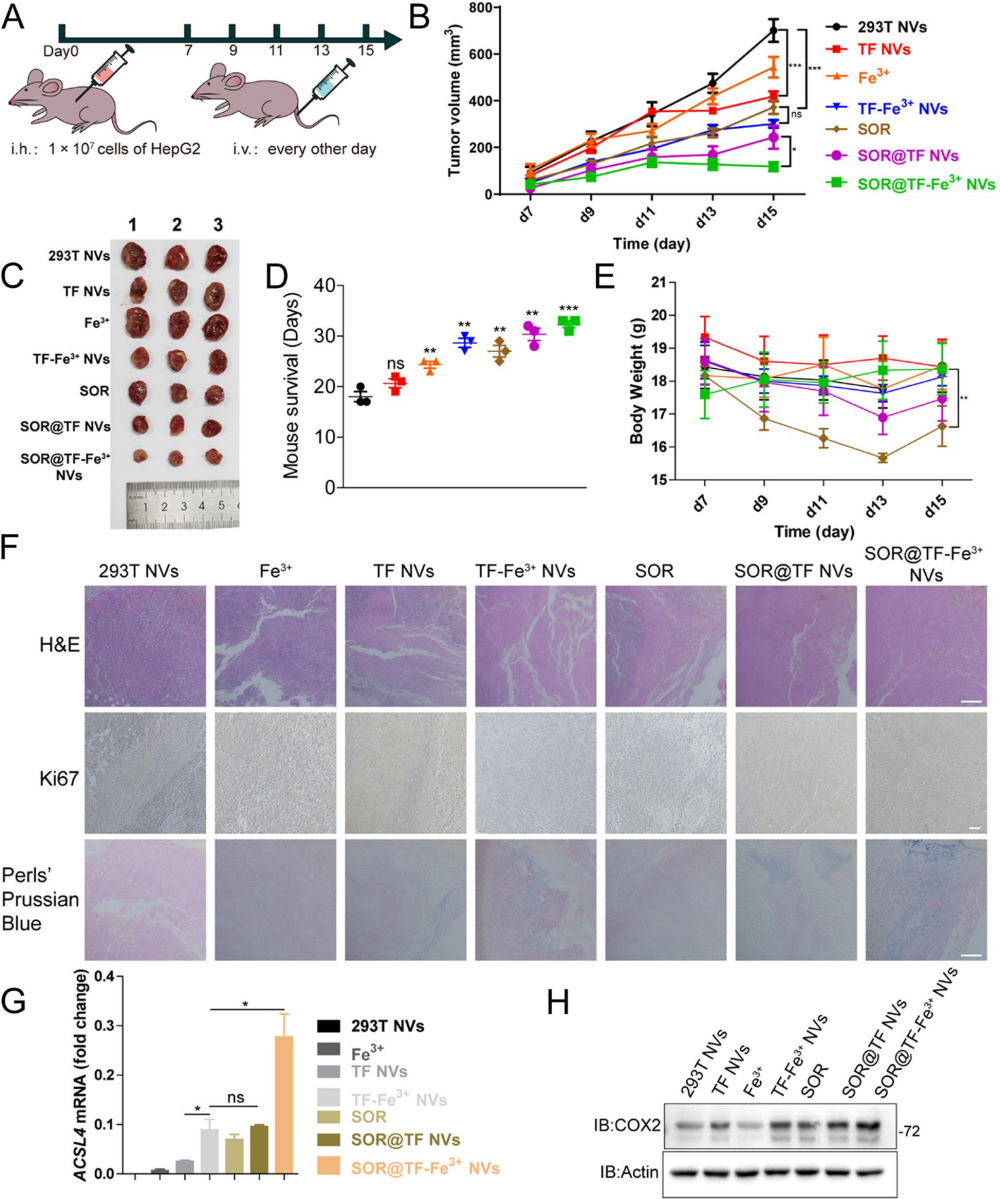
In vivo SOR@TF-Fe^3+^ NVs induced ferroptosis, inhibited the growth of tumor, prolonged the survival of mouse in HCC model. **A** Model diagram of animal experiment and SOR@TF-Fe^3+^ NVs were used for therapy (25 mg NVs/kg). **B** Changes in tumor volume in mice from the beginning of administration (D7) to the end of administration (D15), *n* = 5. **C** Comparison of tumor tissues after administration. **D** Mouse survival curve of another group of mice treated with the same treatment after discontinuation of D16, *n* = 5. **E** Body weight changes of mice during administration, *n* = 3. **F** H&E, Ki67, and Prussian Blue staining of tumor tissue, scale: 100 μm. **G** qPCR analysis of the expression level of ferroptosis indicator *ACSL4* mRNA in tumor tissue,* n* = 3. **H** Western blot analysis of the expression level of ferroptosis indicator COX2 protein in tumor tissues. Data are expressed as mean ± standard error (SEM), ns: no significant, *P ≤ 0.05, **P ≤ 0.01, ***P ≤ 0.001, one-way analysis of variance, ANOVA

In order to demonstrate whether the SOR@TF-Fe^3+^ NVs could inhibit the growth of in vivo tumors, a subcutaneous HepG2 xenograft model in nude mice was established. Once grafted tumors reached an appropriate size (60–70 mm^3^), mice were injected intravenously with either free NVs (25 mg/kg), Fe^3+^ solution (3 mg/kg), TF NVs (25 mg/kg), TF-Fe^3+^ NVs (25 mg/kg), SOR (5 mg/kg), SOR@TF NVs (25 mg/kg), or SOR@TF-Fe^3+^ NVs (25 mg/kg) every other day until 15 days post-graft, before being euthanized and harvested (Fig. 5A). Firstly, H&E staining and blood cell counts showed no obvious damage of NVs treatments to any main organ or hematopoietic systems (Figure S8A, B, Supporting Information). Consistent with our in vitro investigation, we found that SOR@TF-Fe^3+^ NVs significantly suppressed HCC tumor growth (Fig. 5B and C) and extended the survival time of tumor-bearing mice (Fig. 5D). This was more than the TF-Fe^3+^ NVs and SOR treatments alone, suggesting a good synergistic therapeutic effect. Intriguingly, treatment with TF NVs also significantly retarded tumor growth compared to the 293T NVs treatment, which may be due to the binding of serum iron ions to TF NVs which then induce ferroptosis (Fig. 5B, C and E). It is worth noting that cell membrane encapsulated SOR was observed to significantly limit the weight loss of animals compared to the free SOR treatment group (Fig. 5E), indicating that TF-labeled NVs encapsulation substantially reduced the toxic side effects of the SOR drug, very likely as a result of enhanced targeting. In addition, the staining of Ki67 and Prussian Blue revealed clear inhibition of cell proliferation and increased intracellular iron accumulation in the TF NVs-, TF-Fe^3+^ NVs-, SOR-, SOR@TF NVs- and SOR@TF-Fe^3+^ NVs-treated tumors compared to the 293T NVs- and free Fe^3+^-treated groups (Fig. 5F). Of these, the most striking performance was observed in the SOR@TF-Fe^3+^ NVs treated group. Unsurprisingly, the levels of ferroptosis-related biomarkers ACSL4 and COX2 were most significantly elevated in SOR@TF-Fe^3+^ NVs-treated tumor cells, as determined by qPCR and Western blotting (Fig. 5G and H, for gel source data, see Figure S9, Supporting Information).

To further confirmed our hypothesis, an another in situ model of mouse liver cancer was established (Figure S10A, Supporting Information). Nude mice were treated as described in the method until two weeks post-operation. The body weight of mice was smooth changed and showed no significant side effects from the treatment of NVs (Figure S10B, Supporting Information). At the endpoint of the experiment, isolated livers as showed in Figure S10C confirmed the smaller size of tumors in SOR@TF-Fe^3+^ NVs group than that in free NVs group. Metastatic nodules and liver enlargement were also observed in the treatment of free NVs. In contrast, SOR@TF-Fe^3+^ NVs treatment was able to significantly improve these phenomena. Moreover, images and weight examination also revealed a neoplastic enlargement of the spleen under the treatment of free NVs (Figure S10D, Supporting Information). While the size and weight of spleen in SOR@TF-Fe^3+^ NVs group were similar with untreated group (NC). These results suggested that SOR@TF-Fe^3+^ NVs could suppress the growth of *in-situ* liver cancer by inducing ferroptosis, as detected in subcutaneous HepG2 xenograft model.

Taken together, these data revealed that TFRC-targeted TF-Fe^3+^ NVs combined with SOR synergistically induced ample ferroptotic responses in TFRC-overexpressing HCC cells, with an enhanced therapeutic effect compared to the single drug treatment.

## Discussion

The most potent and well tolerated chemotherapeutics destroy cancer cells by interfering with the specific molecular mechanisms involved in uncontrolled cell growth and malignant metastasis, all while avoiding healthy tissue. Despite being one of the few FDA-approved treatments for HCC, SOR is only observed to extend the median survival of patients by 3–5 months [38, 39], and long-term use often leads to serious side effects and the development of drug resistance [40]. As noted previously, however, the anticancer activity of SOR is uniquely due to a novel ferroptotic pathway (probably through inhibition of the X_C_¯(SLC7A11) system), with resistance due to insufficient ferroptosis [20, 30]. Therefore, any method which could enhance the activity of SOR through complimentary ferroptotic mechanisms promises an exceptionally effective therapeutic strategy.

In this study, we found significant co-upregulation of X_C_¯(SLC7A11) and transferrin receptors (TFRC) in the HCC samples of patients through analysis of the TCGA database. On the basis of these data, we developed cell membrane-derived transferrin nanovesicles (TF NVs) coupled with Fe^3+^ and encapsulating SOR (SOR@TF-Fe^3+^ NVs) to synergistically promote ferroptosis, even in SOR -resistant HCC cells. This was achieved by targeting two important ferroptotic pathways, a) through marked LIP accumulation by TF-Fe^3+^/TFRC-mediated iron delivery, leading to the enhanced generation of ROS, and b) SOR inhibition of the X_C_¯(SLC7A11)/GPX4 axis, thereby causing a reduction in protective antioxidant activity. As expected, SOR@TF-Fe^3+^ NVs elicited significant ferroptosis, inhibited tumor proliferation, and prolonged the survival rate in both subcutaneous and in situ HCC mice, which was more effective than SOR alone. These results suggest that our targeted dual approach could be an effective combined treatment strategy for HCC.

More broadly, cell membrane-derived nanovesicles exhibit excellent characteristics as biological carriers. Firstly, nanovesicles can be straightforwardly encoded to display complex proteins with the correct native structure. Indeed, in this study TF protein was expressed on the membrane of nanovesicles by genetic engineering, so that the TF-nanovesicles had the ability to actively target transport and enter HCC cells with high TFRC expression. Secondly, cell membrane-derived nanovesicles with lipid bimolecular structure can also serve as drug carriers, with notably improved pharmacokinetic properties. Due to the targeting of TF vesicles, the distribution of SOR in normal tissues in the current study was reduced, and so the safety profile was also improved accordingly. Moreover, the effect of nanovesicles passively targets these materials to the liver, with the beneficial effect of increasing the local drug concentration to collocated liver tumors. However, TF NVs is mainly located in the liver and lung organs, which may limit the types of tumors to be treated by this NVs platform. In order to deploy the biomembrane-based nanovesicles platform to other tumor types such as renal tumors, it may be necessary to modify NVs with targeting molecules that specifically target renal tumors.

## Conclusion

In conclusion, we have developed a SOR@TF-Fe^3+^ NVs drug, which not only competently induces ferroptosis by acting on two pathways [X_C_¯(SLC7A11)/GPX4 and iron ion transport], but also specially targets TFRC highly-expressed HCC cells. As such, it represents a promising individualized and targeted therapeutic strategy for HCC tumors with high TFRC-expression, but also underscores the utility of the readily modifiable NV platform – a modality which could easily be deployed to other cancer types (such as lung cancer and kidney cancer) by modifying these tumor-specific targeting molecules.

## Supplementary Information

**Additional file 1:** **Figure S1.** The expression level of ferroptosis-related factors in database and *TFRC* mRNA in HCC. **Figure S2.** Effect of *TFRC *knockdown and overexpression models on iron absorption. **Figure S3.** Drug encapsulation rate and iron binding rate of TF NVs. **Figure S4.** HEK293T cells stably expressing TFRC-OFP was detected by qPCR and Western blot.  **Figure S5.** SOR@TF-Fe^3+^ NVs induced ferroptosis in sorafenib-resistant HCC cells. **Figure S6.** Flow cytometry analysis of intracellular ROS content. **Figure S7.** SOR@TF-Fe^3+^ NVs induced ferroptosis in SOR-resistant HCC cells. **Figure S8.** Safety of SOR@TF-Fe^3+ ^NVs.** Figure S9.** Gel source images for Western blots. **Figure S10. **SOR@TF-Fe^3+^ NVs inhibited the growth of tumor in *in-situ *model of mouse liver cancer. **Supplementary Table 1.** The sequences of primers used for RT-qPCR.

## Data Availability

The datasets used and/or analyzed during the current study are available from the corresponding author on reasonable request.

## Abbreviations

ACSL4: Long-chain-fatty-acid-CoA ligase 4
COX2: Cyclooxygenase-2
DCFH-DA: 2,7-Dichlorodi-hydrofluorescein diacetate
Nec: Necrostatin-1
Fer-1: Ferrostatin-1
FPN: Ferroportin
GPX4: Glutathione peroxidase 4
GSH: Glutathione
H&E: Hematoxylin-eosin staining
HCC: Hepatocellular carcinoma
LIP: Labile iron pool
MDA: Malondialdehyde
NVs: Nanovesicles
ROS: Reactive oxygen species
SEM: Standard error of mean
SLC7A11: Solute carrier family 7 member 11
SOR: Sorafenib
TCGA: The cancer genome atlas
TF: Transferrin
TFRC: Transferrin receptor
TMRE: Tetramethylrhodamine ethyl ester perchlorate
Z-VAD: Z-VAD-FMK
