# Supplementary material for: Fe3+-binding transferrin nanovesicles encapsulating sorafenib induce ferroptosis in hepatocellular carcinoma
Source: Biomater Res. 2023 Jul 1;27:63. doi: 10.1186/s40824-023-00401-x (PMC10314404; doi:10.1186/s40824-023-00401-x)
Supplement: Supplementary file 1 — Additional file 1: Figure S1. The expression level of ferroptosis-related factors in database and TFRC mRNA in HCC. Figure S2. Effect of TFRC knockdown and overexpression models on iron absorption. Figure S3. Drug encapsulation rate and iron binding rate of TF NVs. Figure S4. HEK293T cells stably expressing TFRC-OFP was detected by qPCR and Western blot. Figure S5. SOR@TF-Fe3+ NVs induced ferroptosis in sorafenib-resistant HCC cells. Figure S6. Flow cytometry analysis of intracellular ROS content. Figure S7. SOR@TF-Fe3+ NVs induced ferroptosis in SOR-resistant HCC cells. Figure S8. Safety of SOR@TF-Fe3+ NVs. Figure S9. Gel source images for Western blots. Figure S10. SOR@TF-Fe3+ NVs inhibited the growth of tumor in in-situ model of mouse liver cancer. Supplementary Table 1. The sequences of primers used for RT-qPCR. [file 40824_2023_401_MOESM1_ESM.docx]

**Fe^3+^-binding transferrin nanovesicles encapsulating sorafenib induce ferroptosis in** **hepatocellular carcinoma**

**Youmei Xiao^a,†^, Zhanxue Xu^a,b,†^, Yuan Cheng^c,†^,** **Rufan Huang^a^, Yuan Xie^a^, Hsiang-i Tsai^d^,** **Hualian Zha^a^, Lifang Xi^a^, Kai Wang^a^, Xiaoli Cheng^e^, Yanfeng Gao^a^, Changhua Zhang^f,^*, Fang Cheng^a,^*, Hongbo Chen^a,^***

*^a^ School of* *Pharmaceutical Sciences (Shenzhen), Shenzhen Campus of Sun Yat-sen University, Shenzhen 518107, Guangdong Province, China.*

*^b^ Department of Pharmacy, The Seventh Affiliated Hospital of Sun Yat-Sen University, Shenzhen 518107, Guangdong Province, China.*

*^c^ Department of Hepatobiliary Surgery II, ZhuJiang Hospital, Southern Medical University, Guangzhou 510280, Guangdong Province, China.*

*^d^ Department of Medical Imaging, The Affiliated Hospital of Jiangsu University, Zhenjiang 212001, Jiangsu Province, China.*

*^e^* *Department of Pharmacy, Shenzhen Bao'an Maternal and Child Health Hospital, Shenzhen 518133, Guangdong Province, China.*

*^f^ Center for Digestive Disease, The Seventh Affiliated Hospital, Sun Yat-sen University, Shenzhen 518107, Guangdong Province, China.*

**^1^** Youmei Xiao，Zhanxue Xu and Yuan Cheng contributed equally to this manuscript.

*Corresponding author: Hongbo Chen, Fang Cheng and Changhua Zhang,

Email: chenhb7@mail.sysu.edu.cn (H. Chen), chengf9@mail.sysu.edu.cn (F. Cheng), [zhchangh@mail.sysu.edu.cn](mailto:zhchangh@mail.sysu.edu.cn) (C. Zhang).

**Supplementary Figures**


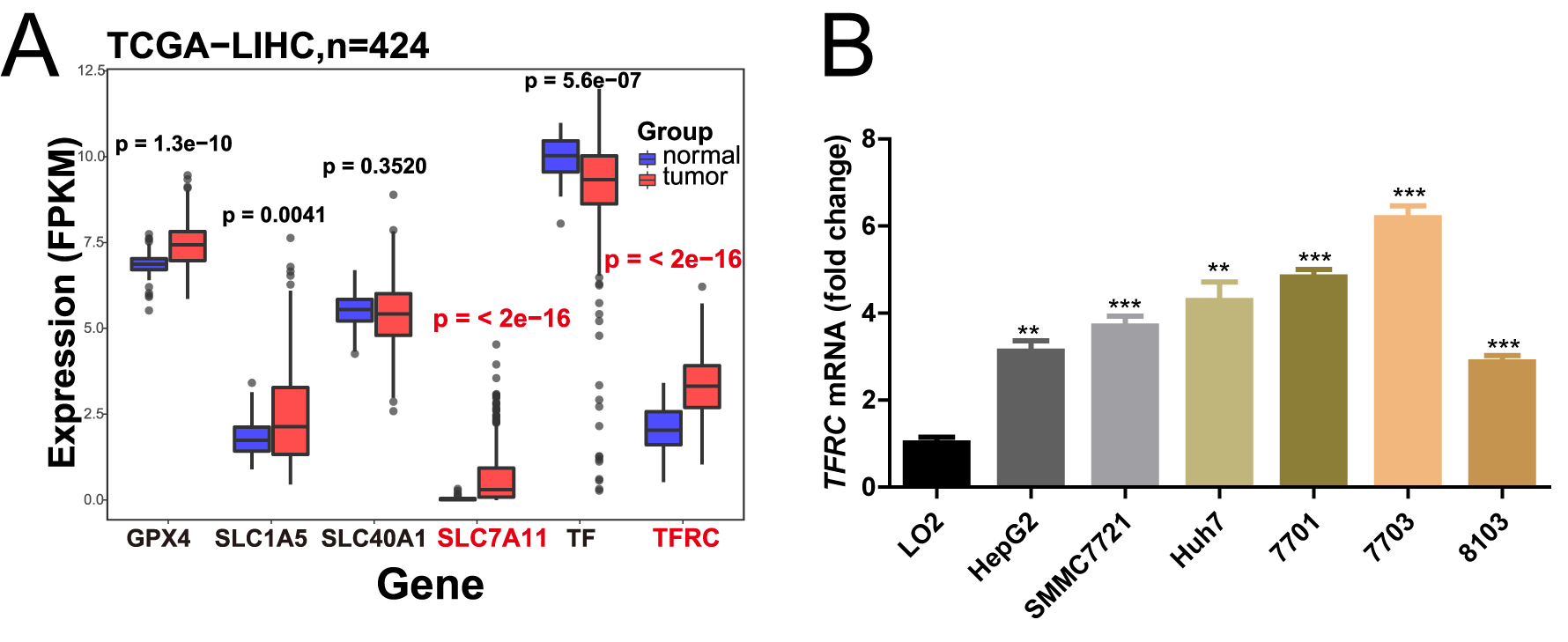


Figure S1. The expression level of ferroptosis-related factors in database and *TFRC* mRNA in HCC. (A) The expression of ferroptosis-related factors between HCC patients (374 cases) and healthy liver tissue samples (50 cases) was analyzed by TCGA. (B) RNA was extracted from HepG2, SMMC7721, Huh7, 7701, 7703 and 8103 cells, and the expression level of *TFRC* mRNA was detected by qPCR.


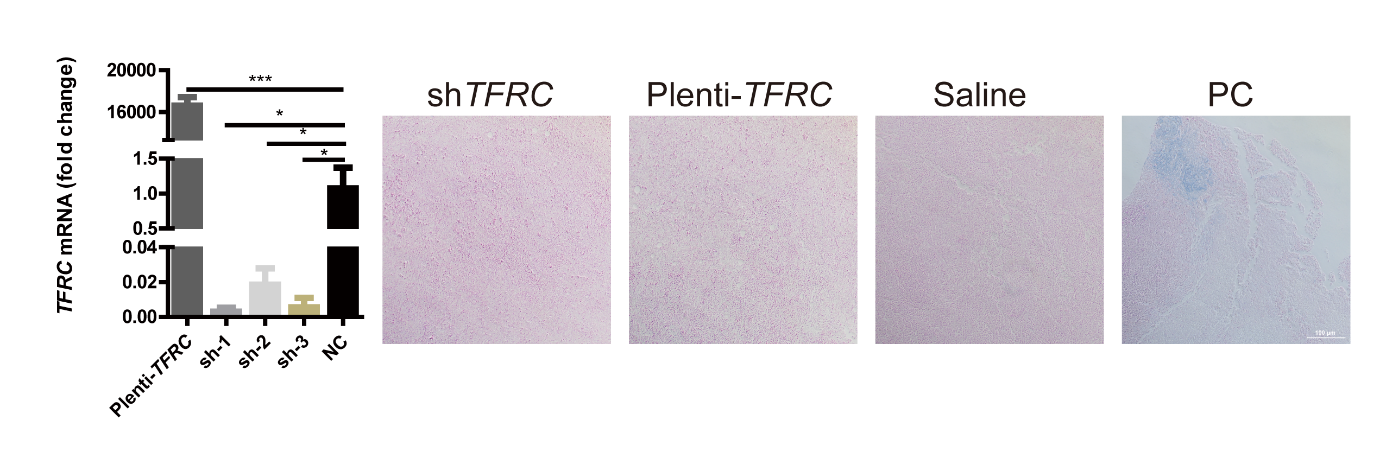


Figure S2. Effect of *TFRC* knockdown and overexpression models on iron absorption. (A) RNA was extracted from overexpressed *TFRC* (Plenti *TFRC*), knockdown *TFRC* (sh-1, sh-2, sh-3) and normal HepG2 cells, respectively, and the *TFRC* mRNA level of cells was detected by qPCR. Data are expressed as mean ± standard error (SEM), *n* = 3. * *P* < 0.05, *** *P* < 0.001. (B) On the 10th day of modeling, the tumor tissues were removed for section analysis with Prussian blue staining. Normal HepG2 cell model was obtained on Saline, and PC was the positive control which 3 mg/kg iron ion was intravenously injected with the next day. Scale bar: 100 μm.


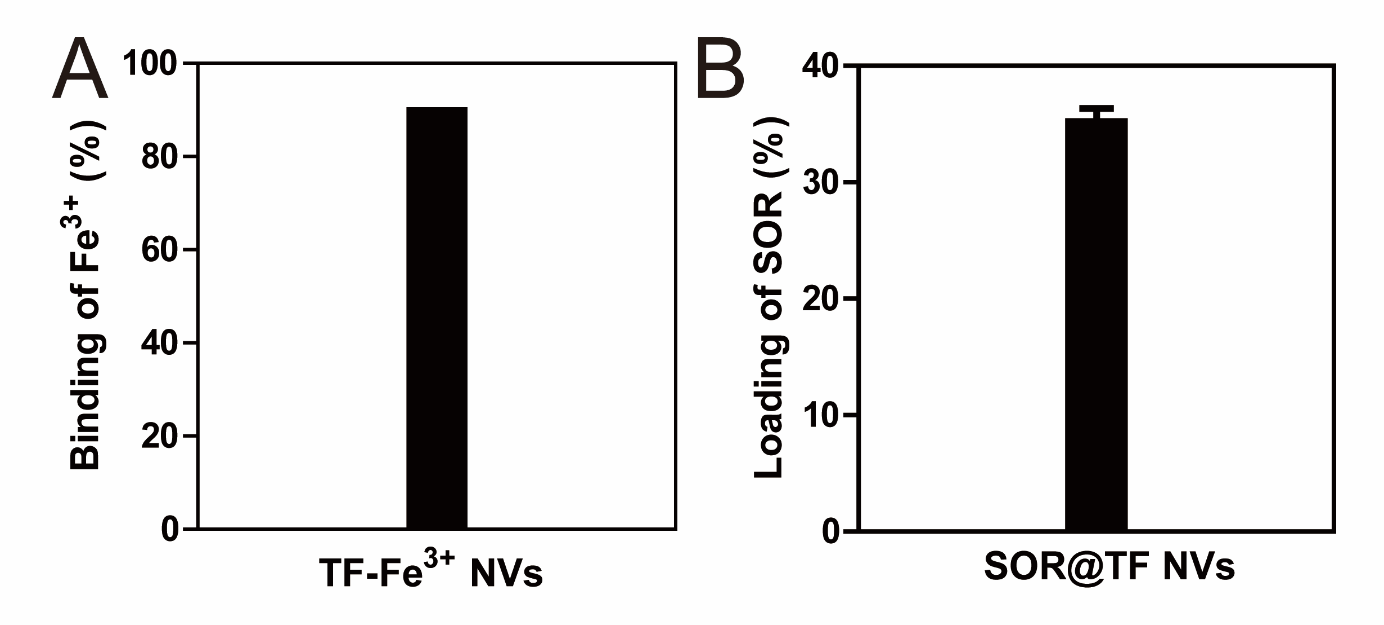


Figure S3. Drug encapsulation rate and iron binding rate of TF NVs. (A) Iron ion binding rate of TF NVs at 37℃ incubations for 24 h, *n* = 3. (B) Encapsulation rate of sorafenib in TF NVs electrical subcontract, *n* = 3.


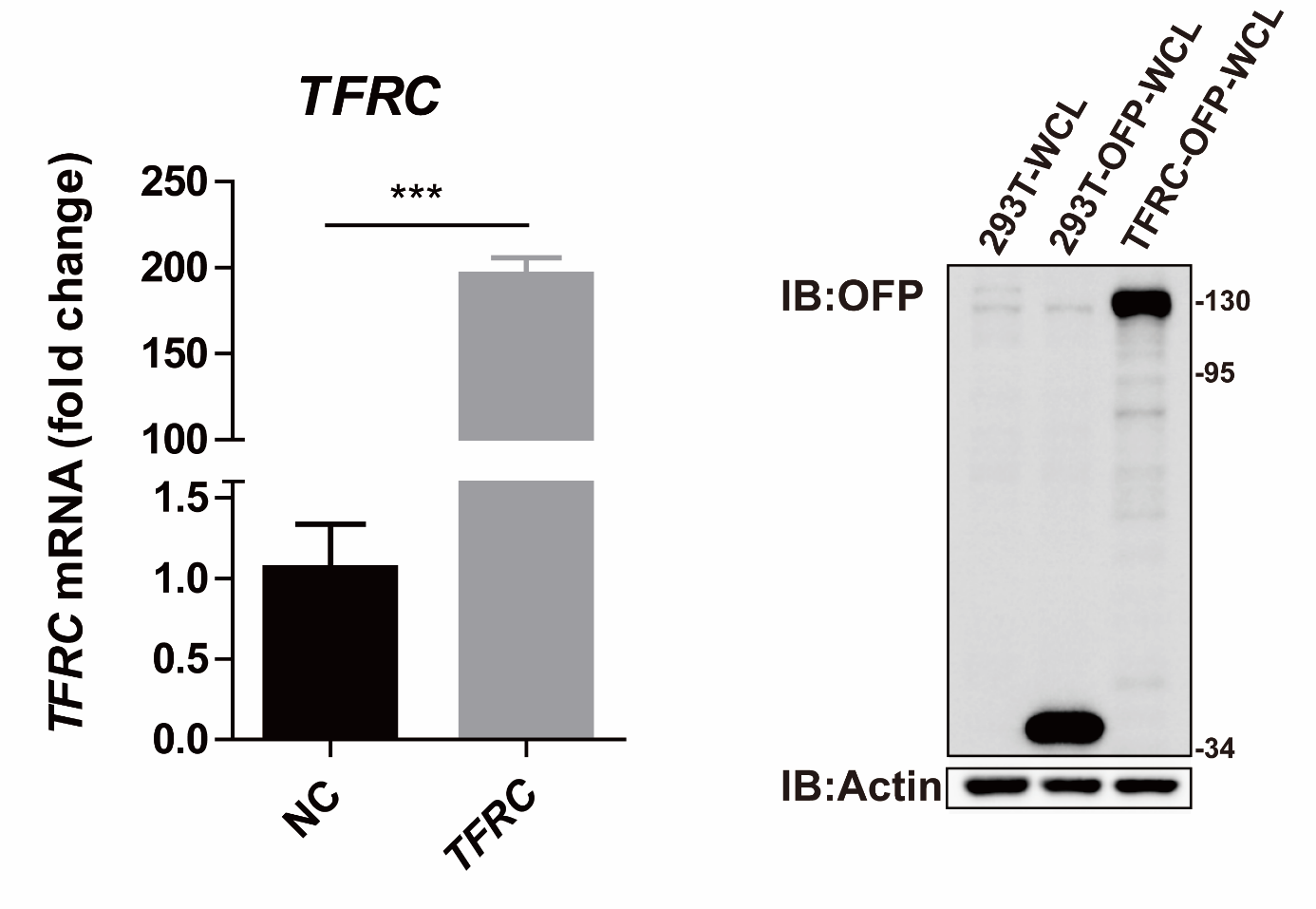


Figure S4. HEK293T cells stably expressing TFRC-OFP was detected by qPCR and Western blot.


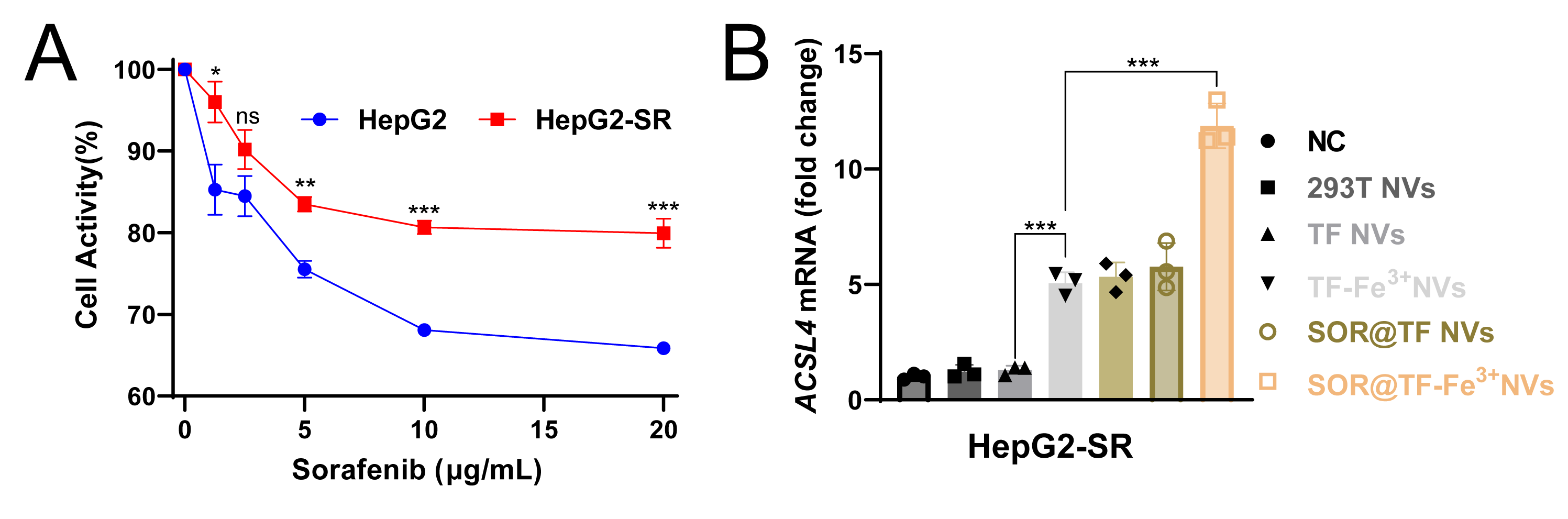


Figure S5. SOR@TF-Fe^3+^ NVs induced ferroptosis in sorafenib-resistant HCC cells. (A) The sorafenib-resistant HepG2 cells (named HepG2-SR) and parental HepG2 were incubated with gradient concentration of sorafenib for 24 h. Cell activity (%) was compared with the corresponding untreated cells. (B) qPCR was used to analyze the expression of *ACSL4* mRNA of HepG2-SR cells. Data are expressed as mean ± standard error (SEM), *n* = 3, ns: no significant, **P* ≤ 0.05, ***P* ≤ 0.01, ****P* ≤ 0.001, one-way analysis of variance, ANOVA.


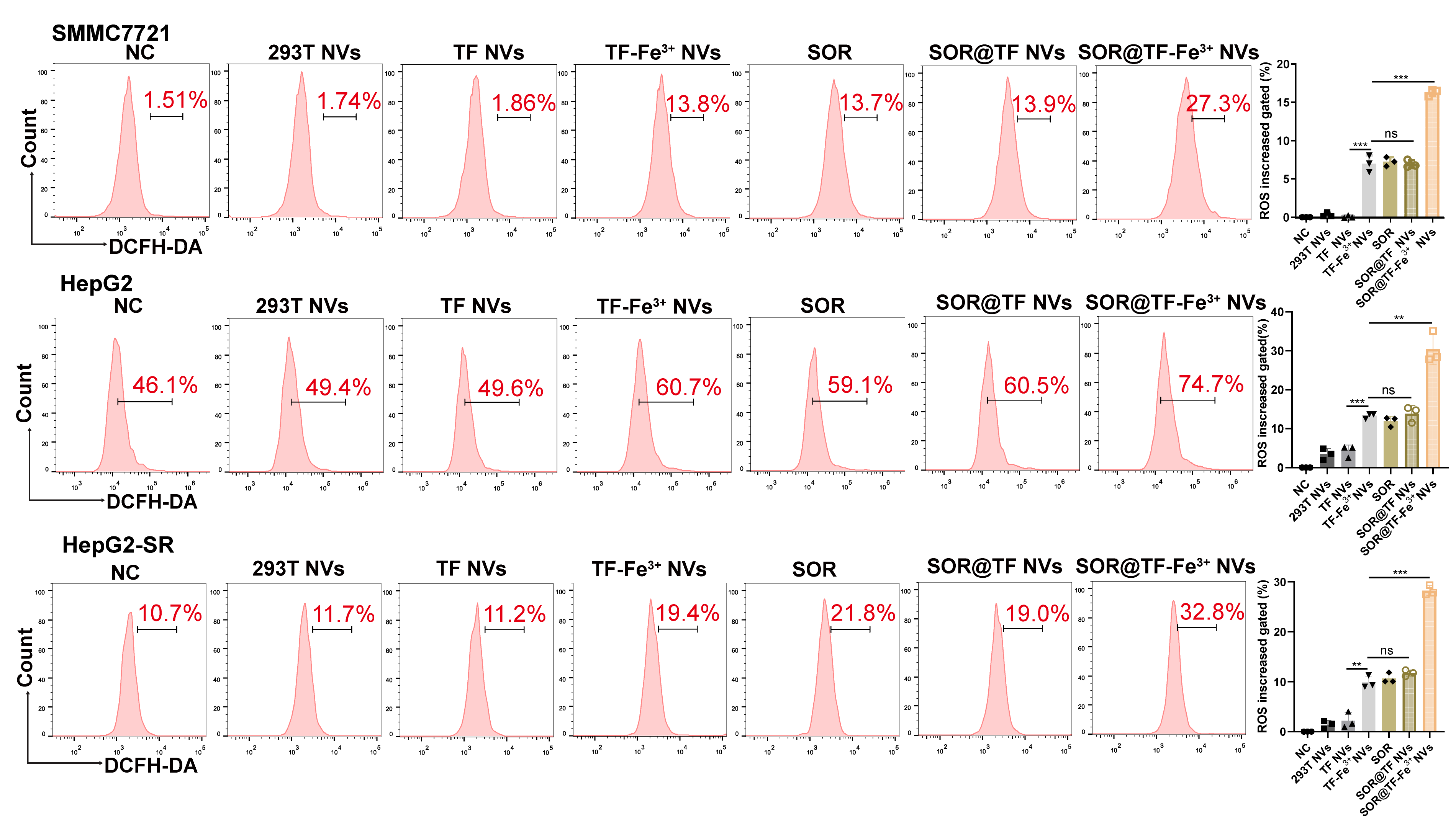


Figure S6. Flow cytometry analysis of intracellular ROS content were tested in SMMC7721, HepG2 and HepG2-SR cells. On the right is the histogram of curve migration quantitative analysis. Data are expressed as mean ± standard error (SEM), *n* = 3, ns: no significant, ***P* ≤ 0.01, ****P* ≤ 0.001, one-way analysis of variance, ANOVA.


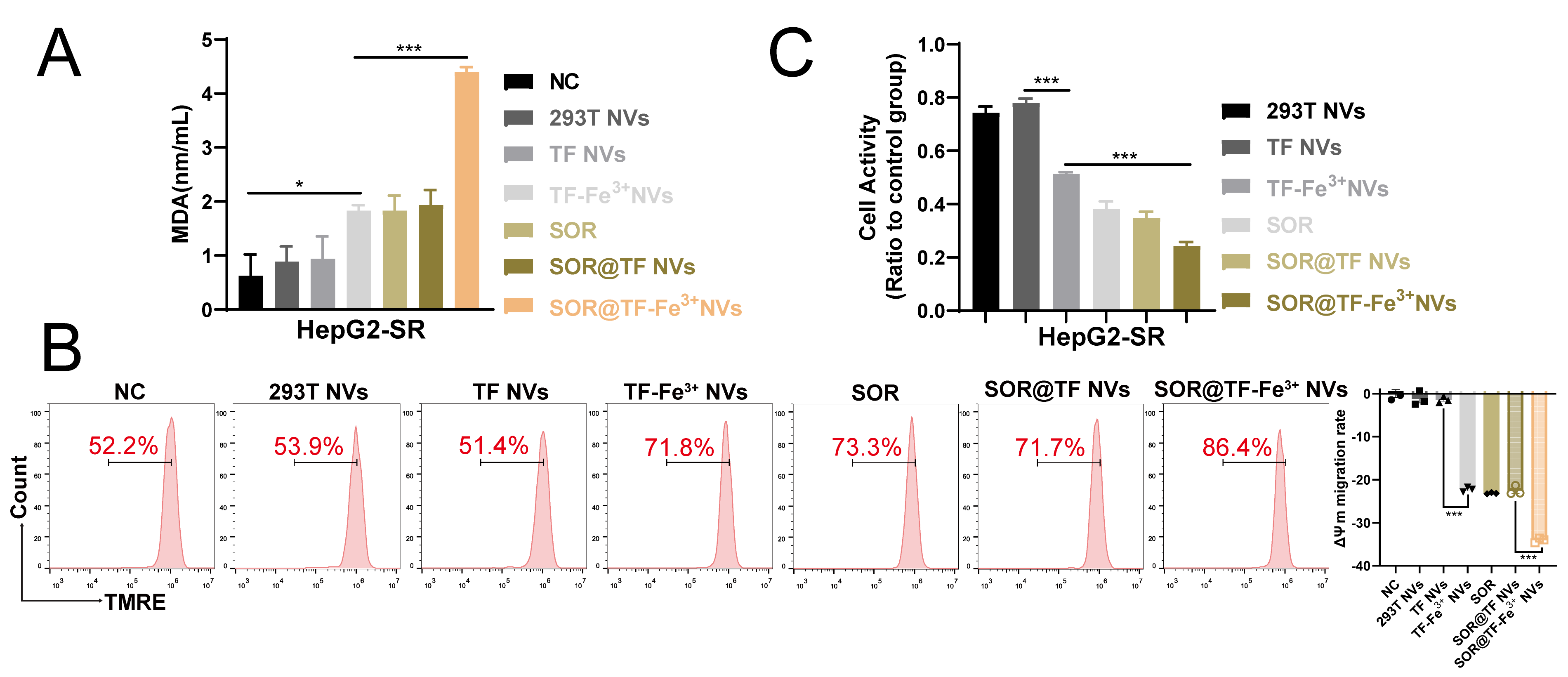


Figure S7. (A) The level of MDA tested by analysis kit after treatment for 24 h. (B) The change of ΔΨm was detected by flow cytometry analysis. On the right is the histogram of curve migration quantitative analysis. (C) The activity changes of HepG2-SR cells after 24 h in each treatment group was detected by CCK-8. Above data are expressed as mean ± standard error (SEM), *n* = 3, **P* ≤ 0.05, ****P* ≤ 0.001, one-way analysis of variance, ANOVA.


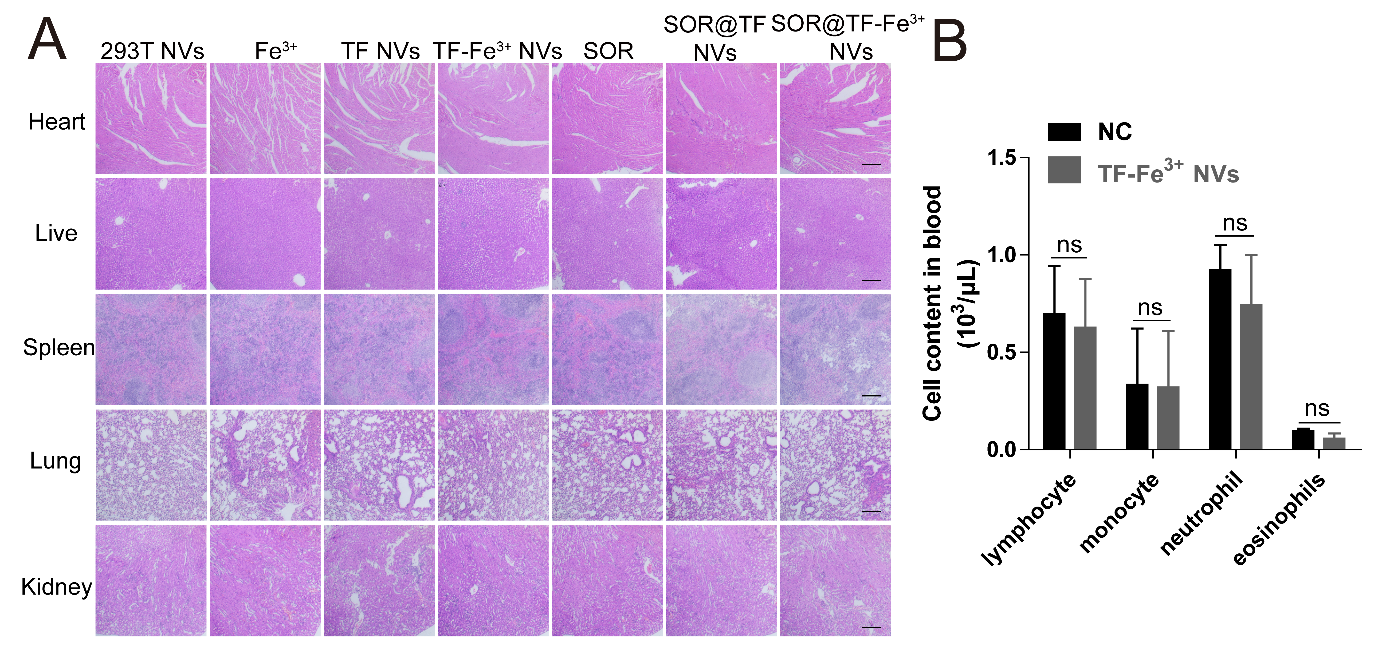


Figure S8. Safety of SOR@TF-Fe^3+^ NVs. (A) H&E staining was used to analyze the damage of heart, liver, spleen, lung, kidney of mice in each treatment group after administration (D15). scale: 100 μm.(B) Complete blood count (CBC) test was used to detect the expression of whole blood cells after administration (D15), *n* = 3, ns: no significant difference.


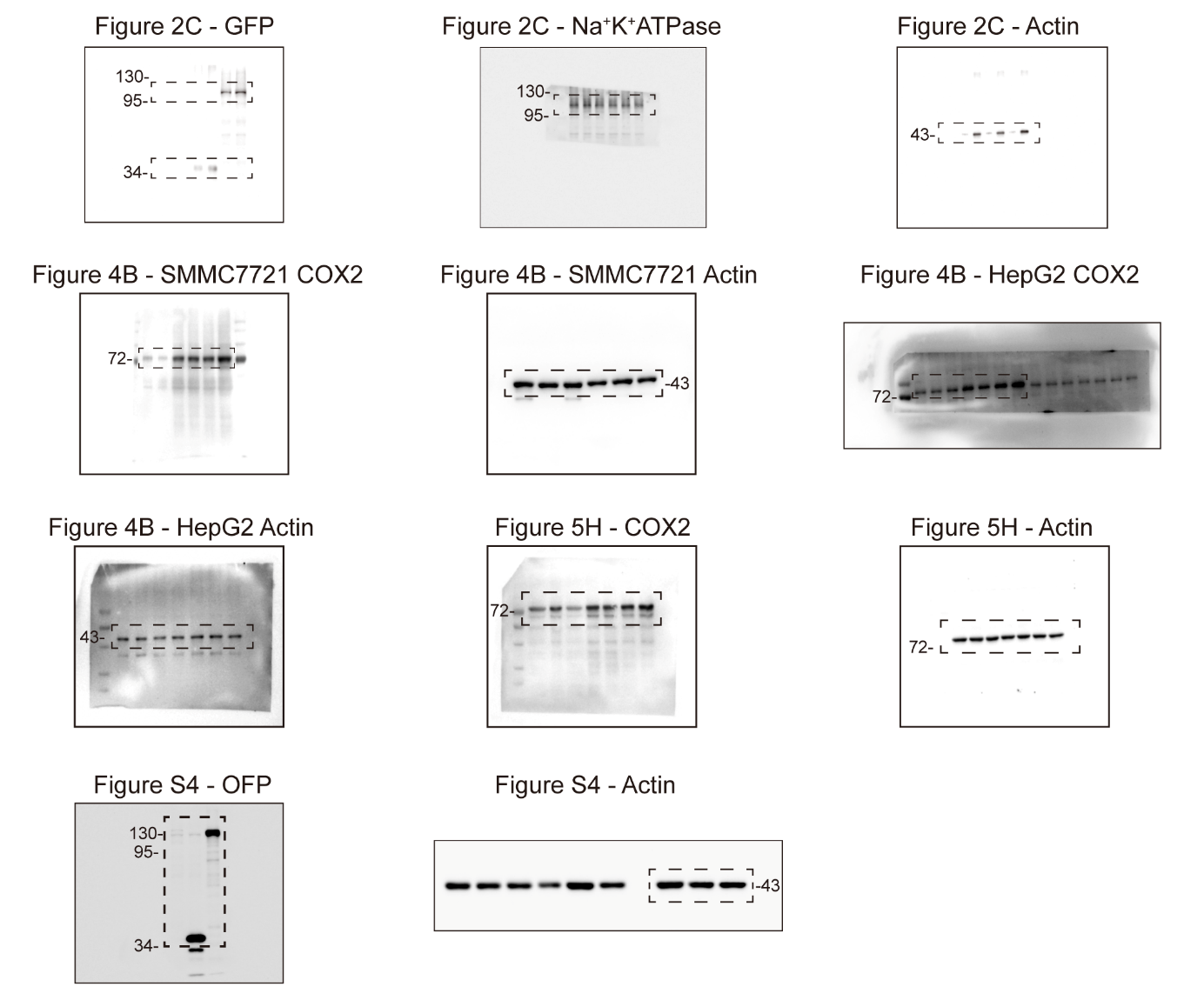


Figure S9. Gel source images for Western blots


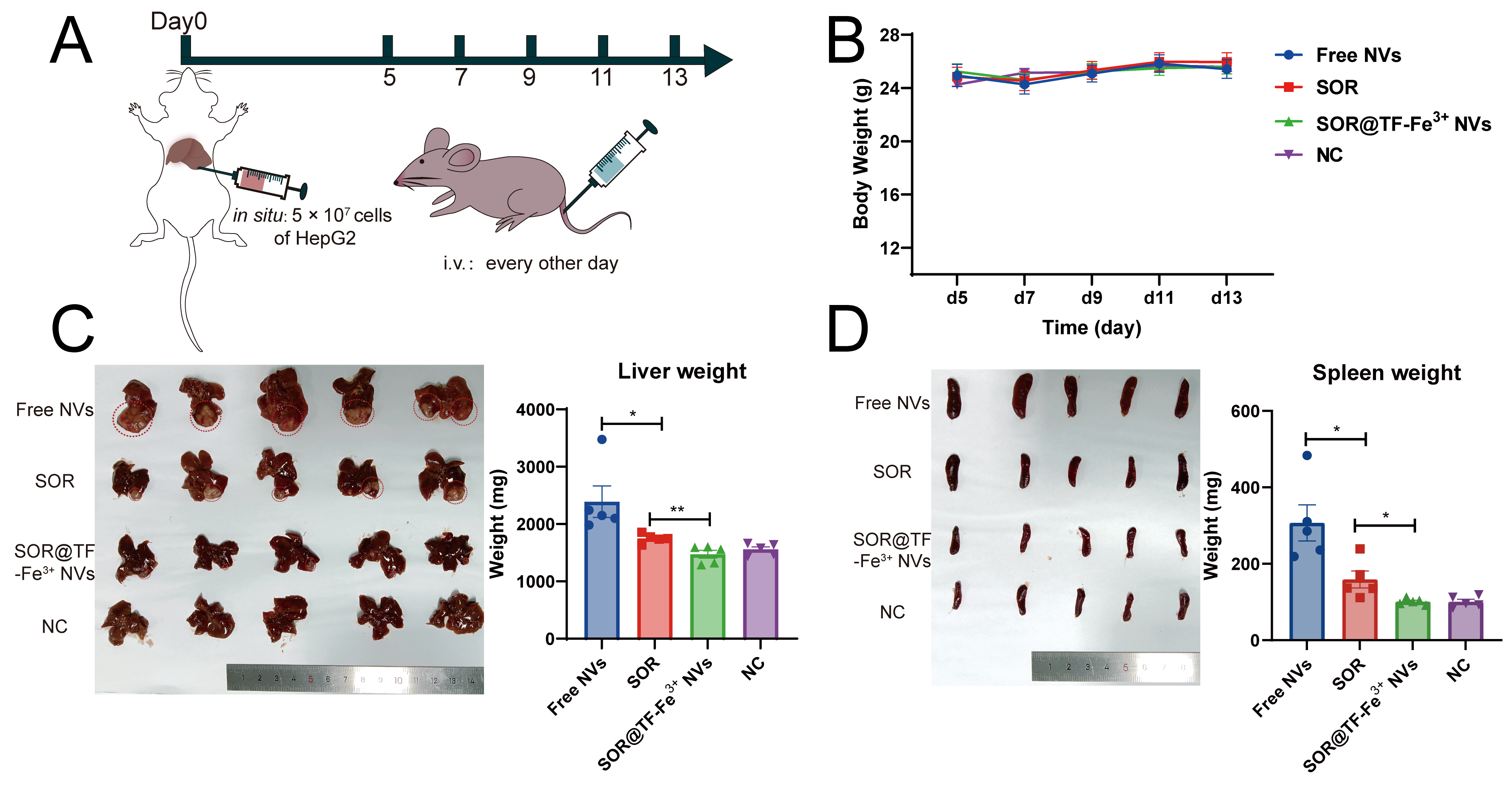


Figure S10. SOR@TF-Fe^3+^ NVs inhibited the growth of tumor in *in-situ* model of mouse liver cancer. (A) Model diagram of animal experiment. (B) The body weight of mouse was monitored every other day. (C) Images and weight of HCC-bearing livers. Red circles indicate HCC tumors. (D) Images and weight of spleens. Above data are expressed as mean ± standard error (SEM), *n* = 5, **P* ≤ 0.05, ***P* ≤ 0.01, one-way analysis of variance, ANOVA.

**Supplementary Table 1**

qPCR primers

| Gene | Forward primer sequence  5’→3’ | Reverse primer sequence  5’→3’ |
| --- | --- | --- |
| Human-*TF* | ATGAACCAGCTTCGAGGCAA | AGAGGTTTACGTGGCTCAGG |
| Human-*TFRC* | ACCATTGTCATATACCCGGTTCA | CAATAGCCCAAGTAGCCAATCAT |
| Human-*ACSL4* | CATCCCTGGAGCAGATACTCT | TCACTTAGGATTTCCCTGGTCC |
| Human-*β-Actin* | CCACACTGTGCCCATCTAC | AGGATCTTCATGAGGTAGTCAGTC |

TF: Transferrin; TFRC: Transferrin receptor; ACSL4: Long-chain-fatty-acid-CoA ligase 4.
